# Alterations in brain fluid physiology during the early stages of development of ischaemic oedema

**DOI:** 10.1186/s12987-024-00534-8

**Published:** 2024-06-10

**Authors:** Stephen B. Hladky, Margery A. Barrand

**Affiliations:** Department of Pharmacology, Tennis Court Rd., Cambridge, CB2 1PD UK

**Keywords:** ATP depletion, Astrocyte swelling, Neuronal swelling, Aquaporin 4, Perivascular spaces, CSF influx, Spreading depolarization, Blood–brain barrier permeability, Donnan effect, Reperfusion

## Abstract

Oedema occurs when higher than normal amounts of solutes and water accumulate in tissues. In brain parenchymal tissue, vasogenic oedema arises from changes in blood–brain barrier permeability, e.g. in peritumoral oedema. Cytotoxic oedema arises from excess accumulation of solutes within cells, e.g. ischaemic oedema following stroke. This type of oedema is initiated when blood flow in the affected core region falls sufficiently to deprive brain cells of the ATP needed to maintain ion gradients. As a consequence, there is: depolarization of neurons; neural uptake of Na^+^ and Cl^−^ and loss of K^+^; neuronal swelling; astrocytic uptake of Na^+^, K^+^ and anions; swelling of astrocytes; and reduction in ISF volume by fluid uptake into neurons and astrocytes. There is increased parenchymal solute content due to metabolic osmolyte production and solute influx from CSF and blood. The greatly increased [K^+^]_isf_ triggers spreading depolarizations into the surrounding penumbra increasing metabolic load leading to increased size of the ischaemic core. Water enters the parenchyma primarily from blood, some passing into astrocyte endfeet via AQP4. In the medium term, e.g. after three hours, NaCl permeability and swelling rate increase with partial opening of tight junctions between blood–brain barrier endothelial cells and opening of SUR1-TPRM4 channels. Swelling is then driven by a Donnan-like effect. Longer term, there is gross failure of the blood–brain barrier. Oedema resolution is slower than its formation. Fluids without colloid, e.g. infused mock CSF, can be reabsorbed across the blood–brain barrier by a Starling-like mechanism whereas infused serum with its colloids must be removed by even slower extravascular means. Large scale oedema can increase intracranial pressure (*ICP*) sufficiently to cause fatal brain herniation. The potentially lethal increase in *ICP* can be avoided by craniectomy or by aspiration of the osmotically active infarcted region. However, the only satisfactory treatment resulting in retention of function is restoration of blood flow, providing this can be achieved relatively quickly. One important objective of current research is to find treatments that increase the time during which reperfusion is successful. Questions still to be resolved are discussed.

## Introduction

Previous reviews in this series [1–5] have covered the principles of regulation of the composition, volume and circulation of brain extracellular fluid (ECF). This review looks at changes in distribution of intracellular fluid (ICF) and ECF in the early stages of ischaemic oedema within the brain. The term oedema refers to *tissue* swelling that results from excess accumulation of watery fluid. This can happen in any part of the body, but this review focusses on that which occurs in brain parenchymal tissue [6, 7].

The barriers to movements of substances between ICF, interstitial fluid (ISF), cerebrospinal fluid (CSF) and blood plasma are sufficiently permeable to water to allow water movements to maintain the osmolality of brain parenchyma closely equal to that in plasma (see appendix B in [1]). Hence even though most of the volume increase occurring during oedema formation results from increased water, in the absence of a change in plasma osmolality there must be accumulation of solutes. There are three possible sources of the additional solutes within the affected region of the parenchymal tissue:

- catabolism of molecules;
- transport from blood across the blood-brain barrier;
- transport from CSF either via perivascular pathways or across the brain surfaces, i.e. across the ependyma lining the ventricles or the pia lining the subarachnoid spaces.

The relative contribution of solutes from these different sources will vary depending on the nature of the oedema, its location and the stage of its development. For example, new osmoles from catabolism may be dominant in regions of necrosis following contusion [8–11] because breakup of proteins and other macromolecules could easily generate enough small molecules to double the amount of solute in a region.

Where oedema is localized, the surrounding regions can usually deform to accommodate the swelling without compromising the local blood flow or disrupting connections between cells. Hence localised oedema is regarded by some as a sign rather than a cause of further development of pathology (see appendix A). However, there must be limits to the degree of swelling. The sum of the volumes of blood, ECF and ICF, together with the solids (see Fig. 2 of [1]) must add up to the volume enclosed by the skull (the Monro-Kellie dogma, see e.g. [12]). Thus if the sum of ICF and ISF, i.e. the tissue fluid volume, increases, the volumes of one or more of the other components must decrease. The volume of blood is normally about 4% of the total cranial volume and small decreases have little consequence. Somewhat larger changes in brain tissue volume can be accommodated by adjusting outflow of CSF and hence the volume it occupies within the cranium. However, still larger total oedema volumes can develop and these may have serious consequences.

Firstly, the increased volume can increase total pressure which can compress cerebral blood vessels sufficiently to reduce blood flow and produce ischaemia, damage and further oedema.

Secondly, if the volume of the cortex, including both grey and white matter, increases to a critical level, then it may herniate across the tentorium into the space occupied normally by the brain stem (for a diagram see [13]). Similarly, if the total volume of the brain becomes too large, there may be herniation of parts of the hindbrain through the foramen magnum. Either form of herniation may be fatal. From 1966 to 1975 there were 100 admissions to the Mayo Clinic for infarction in the territory of the internal cerebral artery ending with the death of the patient. Of these, 31 died of herniation, 25 within 4 days of admission [14]. Similarly in a European study out of 55 patients with complete middle cerebral artery territory infarction herniation was the cause of death in 43 with 12 patients surviving [15].

### Types of oedema

Oedemas can be classified according to their locations, e.g. peritumoral oedema, or their causes, e.g. ischaemic oedema, or most usefully for present purposes their mechanisms. Traditionally, four types of mechanism have been recognised: osmotic, cytotoxic, vasogenic and periventricular. To these should be added the oedema associated with hemorrhage and hematomas. As a result of the early release of biochemically active products into the tissue, this type of oedema is sufficiently different even in its early stages as to warrant a separate review. This has been provided recently by Wan et al. [16]).

Cerebral oedema in which fluid accumulates primarily intracellularly can be experimentally produced very rapidly (minutes) by reducing the osmolality of the blood perfusing the brain. Water then enters into the tissue, moving up the gradient of osmolality (i.e. down its own gradient of chemical potential) across the intact blood–brain barrier and the membranes of the parenchymal cells. This is called **osmotic oedema**.

The distinction between **cytotoxic oedema** and **vasogenic oedema** was introduced by Klatzo [17]. Cytotoxic oedema arises from excess accumulation of solutes within cells. Vasogenic oedema arises from break-down of the blood–brain barrier. There have been a number of suggestions for amendment to this terminology, e.g. that of Betz (see e.g. [7]) who offered the name ‘intact-barrier’ in place of ‘cytotoxic’ and ‘open-barrier’ instead of ‘vasogenic’ and more recently that of Simard et al.[18].^1^ Despite this, Klatzo’s terminology still shapes most discussion. The best example of cytotoxic oedema is the **ischaemic oedema** seen in stroke prior to gross disruption of the blood–brain barrier. The best clinical example of vasogenic oedema is **peritumoral oedema** where the vasculature supplying the tumour is modified and has properties similar to that supplying peripheral tissues. **Periventricular oedema**, which is observed in hydrocephalus and arises from changes in the structure of the parenchyma adjacent to the ventricles, is discussed in Sects. 8.2.1 and 8.2.2.3.1 of [1].

This review is concerned mainly with physiological mechanisms and so will discuss ischaemic oedema with emphasis primarily on events during the first nine to twelve hours immediately after the challenge that provoked the oedema. The review by Jiang et al. [23] covers events important in the longer term changes that take place over days. The long term responses to trauma and the oedema that it causes are considered in the literature of neuropathology, see e.g. [24].

### Cytotoxic oedema

With cytotoxic oedema, the initiating event is a disturbance of metabolism in the cells of the brain parenchyma. Additional osmotically active solutes can be produced within the cells directly by altering metabolism and/or indirectly by reducing supply of ATP and thus impairing the ability to exclude ions. The resultant metabolic disturbances lead to cell swelling as water is drawn in from the surrounding ISF. This transfer of fluid does not in itself cause overall parenchymal swelling because the ISF volume decreases [6, 7]. However, ISF volume becomes partially restored (see further discussion in later sections) due to influx of solutes and water from outside the parenchyma either across the blood–brain barrier [6, 17], or from CSF presumably via perivascular spaces [25, 26] (for background see Sect. 5.6 in [3]). This allows further increase in cell volume and results in overall tissue swelling, i.e. cytotoxic oedema.

More generalised oedema in the whole brain can result following global ischaemia produced by cardiac arrest (see Sect. 3.5.3). In this situation, the resultant cessation of blood flow overall stops delivery of O_2_ and glucose, and removal of CO_2_ from tissues and, as described above, such metabolic disturbances will produce cell swelling at the expense of ISF volume. It is possible that there is some restoration of ISF volume from CSF. If or when blood flow is restored, then there is a source of fluid which can be added to ISF and from there into cells [27].

Global ischaemia leads to irreversible loss of vital neurological functions. This typically can occur in less than 10 min following cardiac arrest. Most neurons survive if blood flow is restored after substantially longer periods but loss of function possibly depends on selective neuronal loss under ischaemic conditions (see e.g. [28, 29]). Selective neuronal loss also occurs in focal ischaemia (see Sects. 4.1 and 5) but long-term functional recovery may occur because surviving portions of the brain can compensate.

### Vasogenic oedema

With vasogenic oedema, the initiating event is a failure, opening or defect in the blood–brain barrier. This will allow solutes, importantly NaCl and plasma proteins, and water to enter the brain parenchyma. The gradients driving entry may be hydrostatic pressure or the resultant of hydrostatic and colloid osmotic pressure depending on the size selectivity of the barrier opening. This results in tissue swelling.

### Other types of swelling

Some forms of tissue swelling are customarily excluded from being called oedema. One important example is brain tissue swelling resulting from vascular engorgement. At least according to Marmarou et al. [30] this latter type of swelling is not regarded as brain oedema. Increase in brain volume resulting from tumour growth is also not of itself oedema, but it is often accompanied by oedema of the surrounding brain tissues.

## Cytotoxic oedema associated with ischaemic stroke. Onset

In ischaemic stroke, blockage of arteries or arterioles decreases the blood flow and thus the supply of O_2_ and glucose to a region of the parenchyma. The decrease can be severe but is normally not total, because there is some supply of blood via collaterals. It is conventional to think of an affected region as divided into a **core** in which neurons and other cells are irreversibly damaged and a **penumbra** in which the cells are still viable. Immediately after the reduction in blood flow the core includes areas of grey matter in which the flow is reduced from a normal value >  ~ 50 mL min^−1^ / (100 g) [31, 32] to <  ~ 10 mL min^−1^ / (100 g) [32–39]. In practice these cells cannot be rescued and will become part of the infarct because attempts at reperfusion cannot be made quickly enough, (see e.g. [29, 40, 41]).

**Fig. 1:**
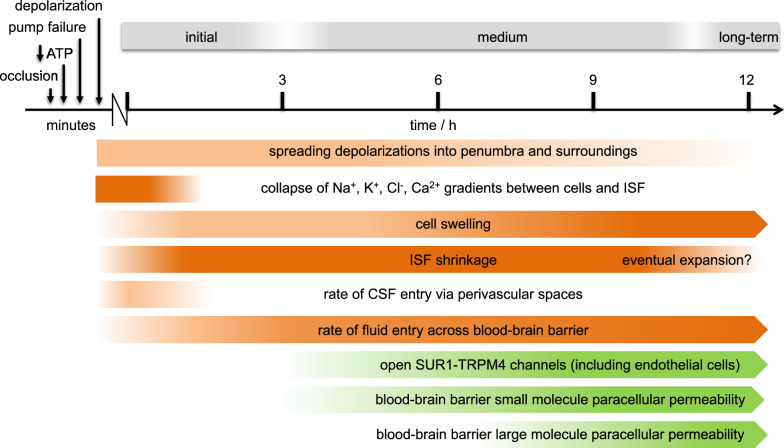
Timeline of changes in the core in the first 12 h of cortical ischaemic oedema. In less than 5 min of ischaemia, intracellular ATP concentrations in the core fall, Na^+^ and Ca^2+^ pumps fail, neurons become depolarized and release K^+^ which is is transferred from neurons to astrocytes. After 5 min (as shown in orange) it becomes evident that: there are spreading depolarizations that extend into the penumbra; ion gradients collapse; both neurons and astrocytes swell at the expense of ISF volume. The combined volume occupied by ISF and cells increases initially as CSF enters enlarged perivascular spaces and subsequently as NaCl and water enter the parenchyma across the blood–brain barrier. The rate of swelling is sustained by changes in blood–brain barrier properties (as shown in green). These include opening of SUR1-TRPM4 channels allowing Na^+^ to pass through endothelial cells and into astrocyte endfeet (see Sects. 3.5.2 and 4.2). There are also conformational changes in the tight junctions between endothelial cells increasing paracellular permeability of the endothelial cell layer (see Sect. 4.2.2). At first this allows small molecules to pass through and eventually large molecules and cells to enter as well. Gross failure of the blood–brain barrier occurs within a day. At longer times than shown there is generalised disintegration both of extracellular matrix and of all cell types in the core

A timeline of changes occurring in the core is shown in Fig. 1.

The surrounding penumbra is functionally defined as the region in which neurons are electrically silent^2^ but still have a nearly normal membrane potential [42]. Blood flow in this region is typically greater than 12 mL min^−1^ / (100 g) but less than 18 mL min^−1^/(100 g) ([32, 36, 37, 39]). The core expands over time (see Fig. 1) at the expense of the penumbra, but this process is slow enough that cells in the penumbra can be rescued by timely reperfusion. The pre-rescue perfusion level threshold required to allow rescue to be possible increases with the delay before reperfusion [36].

## Initial events in the development of ischaemic oedema

The processes involved in the onset of oedema in the core and its initial spread into the penumbra can be summarized as follows:

- **Depolarization of neurons in the core, and neural uptake of Na**^**+**^ **and Cl**^**−**^**, loss of K**^**+**^ a**nd neuronal swelling** (Sect. 3.1). In the core, neurons quickly exhaust their supply of O_2_ and glucose and thus much of their ability to produce the ATP needed to maintain ion gradients and hence membrane potential. They depolarize, take up Na^+^, release somewhat less K^+^, take up Cl^−^ preserving electroneutrality, and swell.
- **Propagation of spreading depolarizations into the penumbra and beyond** (Sect. 3.2). Depolarization of neuronal membranes and greatly increased [K^+^]_isf_ in the core trigger spreading depolarizations that propagate into the penumbra and surrounding parenchyma and lead to expansion of the core.
- **Uptake of Na**^**+**^**, K**^**+**^ **and anions and swelling of astrocytes in the core **(Sect. 3.3). Astrocytes respond to the ischaemia by taking up K^+^, Na^+^, HCO_3_^−^, and Cl^−^ and swelling.
- **Reduction in ISF volume by cellular uptake of fluid** (Sects. 3.4). Swelling of neurons and astrocytes occurs by influx of solutes and water from ISF thus reducing ISF volume.
- **Increase in solute content of the parenchyma and development of oedema** (Sect. 3.5). There is influx of solutes from both CSF (Sect. 3.5.1) and blood (Sect. 3.5.2) and production of additional solutes within the parenchyma (Sect. 3.5.3). This increase in parenchymal solute content leads to influx of water and consequential increase in total tissue volume, i.e. oedema develops. Most of the solutes and water from plasma and from CSF that enter ISF continue into the parenchymal cells.
- **Presence of AQP4 in astrocyte endfeet allowing water that crosses the blood-brain barrier to enter directly into the endfeet** (Sect. 3.6).

Detailed analyses of the processes listed above are described in Sects. 3.1 to 3.6.

### Depolarization of neurons in the core and the immediate consequences

**Fig. 2:**
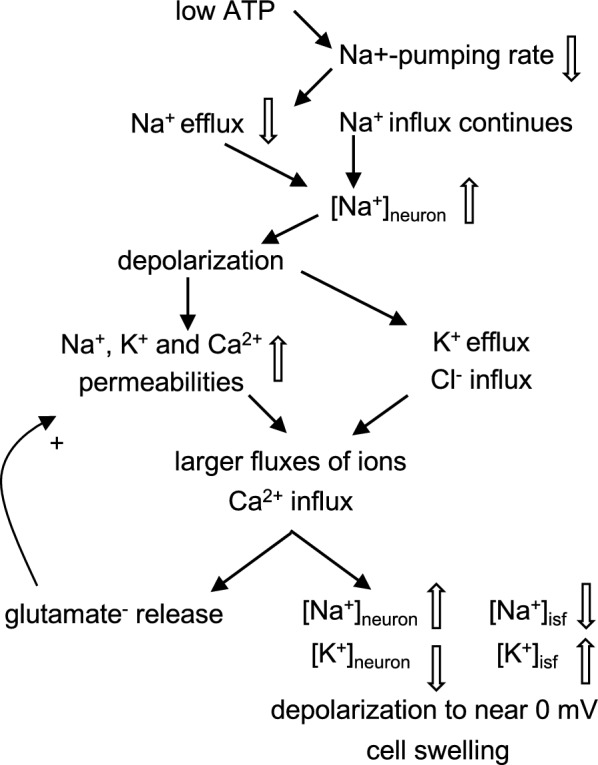
Flow diagram of the ionic changes occurring in neurons at the onset of ischaemia. ATP concentrations fall, active efflux of Na^+^ by the Na^+^-pump is reduced, and Na^+^ influx exceeds its efflux. Hence [Na^+^]_neuron_ increases and [Na^+^]_isf_ decreases and this increase in positively-charged Na^+^ inside the neuron leads to depolarization. This results in K^+^ efflux and Cl^−^ entry with further Na^+^ influx balancing these ion movements to preserve electroneutrality. Other effects of depolarization are increases in permeability (i.e. opening more voltage-sensitive channels) to Na^+^ and Ca^2+^ leading to further increases in [Na^+^]_neuron_ and [Ca^2+^]_neuron_, further depolarization and release of glutamate^−^. Increased [glutamate^−^]_isf_ leads to further increase in the permeability to Na^+^ and Ca^2+^ via glutamate-activated channels. The accompanying decrease in [Na^+^]_isf_ and increase in [K^+^]_isf_ can be by as much as 80 mM (to roughly half of normal) and 40–60 mM (more than ten times normal) respectively. The gain of Na^+^ and Cl^−^ exceeds the loss of K^+^ and the neurons swell at the expense of ISF

At the onset of ischaemia, within the core there is increased reliance on glycolysis to provide ATP.^3^ This anaerobic metabolism reduces *pH* and increases [lactate^−^] (see appendix B). However, despite glycolysis, the ATP production is insufficient to meet the demands of active pumping of Na^+^ and Ca^2+^ leading to changes in ionic concentrations, membrane potential and cell volume as indicated in Fig. 2.

K^+^ efflux is initially via the K^+^ channels normally open at the resting potential while Cl^−^ entry is at least partly via cotransporters (see [44–46]. The increase in [Na^+^]_neuron_ and decrease in [Na^+^]_isf_ reverse the direction of ion movement by Na^+^-glutamate uptake-transporters leading to glutamate release and increase in [glutamate^−^]_isf_ [47] (see Fig. 2). The increase in [Ca^2+^]_neuron_ may also stimulate further release of glutamate^−^ from vesicles.

The influxes of Na^+^ and Cl^−^ into neurons during ischaemia are accompanied by cell swelling indicative of entry of water [48] (for graphic examples of swelling see supplementary files in [45]). The extent of swelling and the routes by which the water enters have been the subject of some controversy. The water permeability of neurons when measured in brain slices appears to be very low which led Andrew et al. [49] to propose that the depolarization resulting from ischaemia opens channels that are water permeable. Alternatively, it has been proposed that the water enters by co-transport with inorganic ions, e.g. via transporters like KCC2 and NKCC1, and co-transport with lactate^−^, e.g. via MCT2 [44]. 

Further aspects of the events involved in the depolarization and swelling of neurons are discussed in appendix C.

### Spreading depolarizations in the penumbra and beyond

**Fig. 3:**
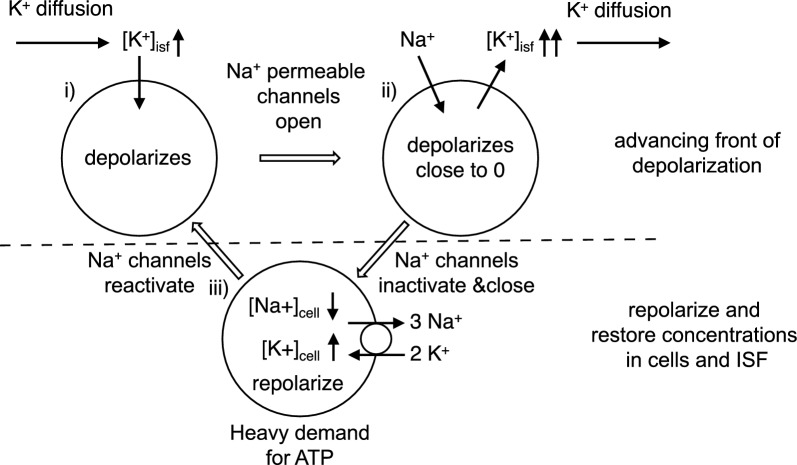
Figure depicting events involved in spreading depolarization. **i** The depolarization of neurons is initiated by diffusion of K^+^ from adjacent already depolarized tissue. This increase in [K^+^]_isf_ leads to influx of K^+^ and to depolarization. **ii** Depolarization then triggers opening of Na^+^ channels and influx of Na^+^ which amplifies the depolarization leading to release of K^+^ which further increases [K^+^]_isf_. Diffusion of K^+^ to adjacent cells propagates the wave of depolarization. **iii** The energy required by neurons to recover from the gain of Na^+^ and loss of K^+^ far exceeds that required for recovery from normal neural activity

The depolarization of neurons and increased [K^+^]_isf_ in the core (see Sect. 3.1) serve as the origin for spreading depolarizations into surrounding regions of the parenchyma^4^ [51–56]. Diffusion of K^+^ from the area of raised [K^+^]_isf_ increases the local concentration of K^+^ in neighbouring regions which drives K^+^ entry via the K^+^ channels already open at rest. This entry is sufficient to initiate depolarization of neurons (see Fig. 3) and in turn to provoke Na^+^ and Ca^2+^ entry leading to much greater depolarization (to ~ 0 mV) and release of K^+^. These changes propagate through the tissue albeit much more slowly than action potentials [43, 57–60].

Although cells in the core region remain depolarized, those in the adjacent penumbral region can repolarize and be subject to further waves of depolarization.^5^ However, repolarization places a heavy burden on ATP consumption and eventually the cells remain depolarized and become part of the permanently damaged core region [48, 53, 60–62]). The metabolic demands imposed by spreading depolarizations are likely to be more important than reduced blood flow in decreasing ATP levels in the penumbra and thus in the expansion of the core [48].

### Uptake of Na^+^, K^+^ and anions by astrocytes and their resultant swelling in the core.

**Fig. 4:**
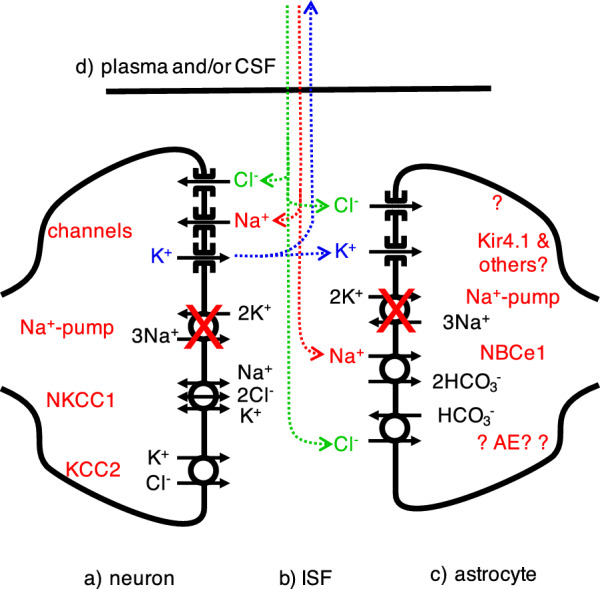
Changes in ion transport between neurons, astrocytes, ISF, CSF and plasma following depletion of ATP in the core. **a** In the neurons, because ATP is depleted, the Na^+^,K^+^- ATPase (the Na^+^- pump) can no longer produce outward movement of Na^+^ to balance its inward movement via channels and other transporters. The net entry of positively charged Na^+^ depolarizes the cell membrane. This depolarization opens further routes for Na^+^ entry and also leads to K^+^ exit via K^+^ channels and to Cl^−^ entry via unspecified channels or cotransporters. The gain of Na^+^ exceeds the loss of K^+^, the net accumulation of cations and Cl^−^ draws in water from ISF and thus the neurons swell. **b** In ISF, as a result of neuronal Na^+^ uptake and K^+^ release, [Na^+^]_isf_ decreases to as low as 60–70 mM whilst [K^+^]_isf_ increases to as much as 40–60 mM. **c** Astrocytes respond to the increase in [K^+^]_isf_ by taking up K^+^ via K^+^ channels so depolarizing their membranes. Na^+^ and HCO_3_^−^ enter via the NBCe1 cotransporter and the associated entry of net negative charge allows further entry of K^+^. Some of the HCO_3_^−^ may exchange with Cl^−^. The astrocytes swell by taking up water from ISF or from perivascular spaces via their AQP4-containing endfeet membranes (see Sect. 3.6 and appendix E for further discussion). The events in **a**, **b** and **c** occur within a few minutes of the onset of ischaemia. d) Also starting immediately but progressing over hours is net entry of Na^+^ (red) and Cl^−^ (green) into the parenchyma from outside, i.e. from CSF and/or plasma. There is also net loss of K^+^ (blue) from the parenchyma to CSF and/or plasma. These ion movements, maintaining electroneutrality, result in a gain of parenchymal solute content, entry of water and thus formation of oedema

The uptake of K^+^ by astrocytes serves to buffer [K^+^]_isf_, i.e. it blunts the increase in [K^+^]_isf_ when K^+^ is released from neurons. Under normal circumstances this is beneficial as it stabilizes the resting potential of neurons, but in the context of ischaemia it serves to delay elimination of K^+^ from the damaged region [63]. The ion transporters involved in the initial responses of both neurons and astrocytes are indicated schematically in Fig. 4. These changes have been described at length by Somjen and his work should be consulted for further background and an introduction to quantitative simulations of the events [43, 64].

Astrocytes have a high resting K^+^ conductance due to the presence of multiple types of channel including prominently Kir4.1 [65–68] (see Fig. 4 and appendix C). As a consequence, the increase in [K^+^]_isf_ will lead to entry of K^+^ and astrocyte membrane depolarization [43, 69, 70]. Further depolarization results if the rate of Na^+^-pumping is reduced as a consequence of reduced ATP.^6^

Only a small amount of K^+^ entry will produce depolarization sufficient to stop further net K^+^ entry unless the K^+^ entry can be accompanied by anions (or efflux of H^+^).^7^ To account for significant accumulation of K^+^ in the astrocytes, several routes for anion entry have been proposed. One is an anion channel [83]. However, resting astrocytes are reported not to have such a channel [84, 85] (but see [69, 86]). It is possible in ischaemic conditions Cl^−^ channels are opened in response to depolarization or to increased [Ca^2+^]_astrocyte_ (for further discussion of anion channels see [85, 87–89]). Another suggestion for an anion entry route is the Na^+^, K^+^, 2Cl^−^ cotransporter NKCC1 [90] but, on current evidence, this transporter is not expressed in mature astrocytes (see appendix C). It is now thought more likely that the anion that enters during ischaemia is HCO_3_^−^ [91–93] mediated by the electrogenic Na^+^, 2 HCO_3_^−^ transporter NBCe1 [83, 94–96] (see Fig. 4).

The net entry of K^+^, Na^+^, HCO_3_^−^, and Cl^−^ described above is accompanied by water and the astrocytes swell [97]. Some of the water entry is directly coupled to transport mediated by NBCe1 [95, 96, 98] and some is likely to move from endothelial cells into astrocyte endfeet via their basement membranes and AQP4 in the blood-vessel-facing membranes of the endfeet (see Sect. 3.6). Further consideration of the responses of astrocytes to ischaemia is given in appendix C.

### Changes in ISF and development of oedema

The processes described in Sects. 3.1 & 3.3 account for parenchymal cell swelling due to uptake of fluid from the adjacent ISF. However, these processes alone would not produce an overall parenchymal tissue swelling because they also produce an initially matching decrease in ISF volume, a decrease which has been observed experimentally using a number of techniques.^8^ This ISF volume decrease is maintained for days in ischaemia. However, the total tissue volume progressively increases on account of the cells continuing to swell, i.e. there is oedema. This is due to an increase in the total amount of solutes and water in cells and in ISF combined. The solutes derive both from entry of ions from outside the tissue and from metabolic production of osmolytes within the tissue as discussed in Sects. 3.5.1 to 3.5.3.^9^

It is possible and convenient to describe separately the processes involved in the transfer of osmoles from ISF to parenchymal cells and those involved in transfers from CSF and plasma to ISF. This stems from the separate locations of the two sets of processes. However, it should be emphasized that these processes are inextricably linked by variations in ISF composition and are not separated in time. Both sets of processes function at the same time to produce the observable cytotoxic oedema [6].

**Fig. 5:**
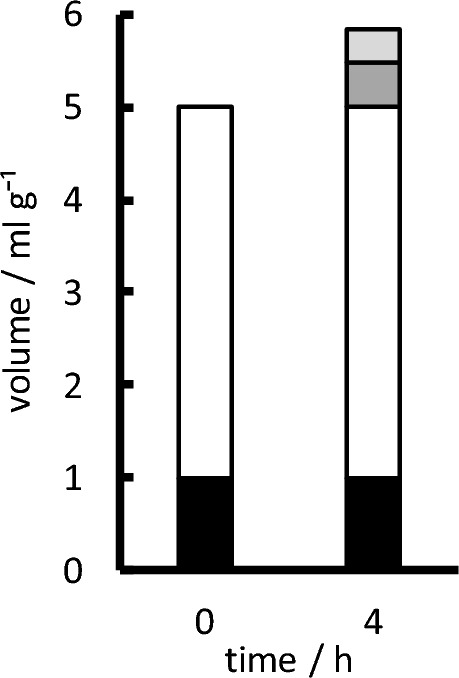
Illustration of the increase in parenchymal tissue volume during development of oedema four hours after onset of MCAO. The heights of the columns indicate volumes per gram of tissue dry weight: tissue solids (black); the initial fluid volume both intra- and extracellular (white); the additional volume resulting from net uptake of ions (dark grey), i.e. NaCl influx, but KCl efflux; and the additional volume (light grey) resulting from metabolic production within the tissue of new osmoles together with the amount of water that maintains nearly constant osmolality, ~ 310 mOsmol L^−1^. The fraction of the volume increase attributable to net uptake of ions is *f*_ionic_ = (net ion gain) _/_ ((increase in volume) x (310 mOsmol L^−1^)) and the fraction attributable to production of new osmoles is 1—*f*_ionic_. In these expressions, the net ion gain is the sum of the Na^+^ and Cl^−^ gains minus the K^+^ loss; and the Cl^−^ uptake is assumed to be equal to Na^+^ gain minus K^+^ loss. Data from appendix D

Oedema formation of the order of 0.8 mL g^−1^ (increase in tissue volume per unit dry weight) has been observed within the first 3 to 4 h in experimental studies using middle cerebral artery occlusion (MCAO) (see Fig. 5 for an indication of the volume changes and appendix D for a compilation of the available data). The increases in total osmolality measured in the parenchyma ([102, 112–114], appendix D) are sufficient to drive the observed water gain (see appendix B in [1]).

### Sources of solutes added to the parenchyma during development of oedema

There are three possible sources of solutes added to the parenchyma during oedema: CSF (Sect. 3.5.1); blood (Sect. 3.5.2); and metabolic production within the tissue (Sect. 3.5.3).

#### *Influx of solutes *via* CSF*

Solutes, primarily NaCl, can enter the parenchyma from CSF either by diffusion across the surfaces of the parenchyma or, as proposed by Thrane et al. [25, 115] and Mestre et al. [26], by diffusion and convection in perivascular spaces. Experimental evidence for CSF inflow during ischaemia comes from studies of the very early changes in solute influx and water content following MCAO [26],^10^.^11^

**Fig. 6:**
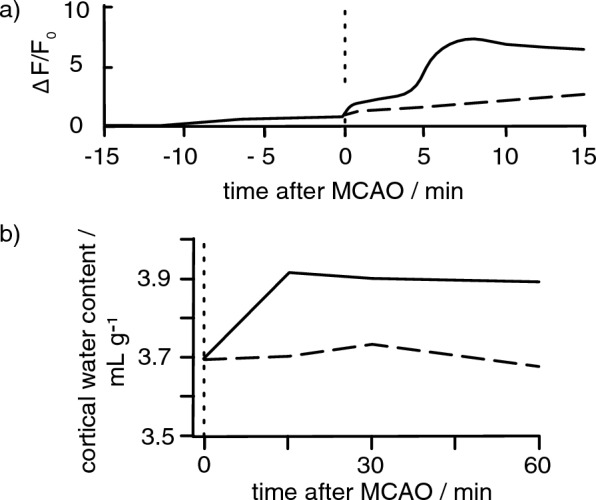
Time courses of intakes of a fluorescent CSF marker a) and of water b) into a brain region following MCAO. **a** Changes in fluorescence from the marker added to CSF in the cisterna magna at t = -15 min, shown for regions on the ipsilateral (solid) and contralateral (dashed) sides. *F*_0_ is the fluorescence at t = 0. **b** Water content of the ipsilateral (solid) and contralateral (dashed) regions shown as mL of water per gram dry weight of tissue. Note that the intake of the fluorescent marker in **a** and of water in **b** appear to be complete within a few minutes. Furthermore, the increase in water content, about 0.2 mL g^−1^ over 15 min, is substantially less than the increases over 3 to 4 h measured by others in different species (see appendix D and Fig. 5). Redrawn and simplified from data in Fig. 1 of Mestre et al. [26]

Mestre et al. showed (see Fig. 6):

- in the first 15 min there was an increase in parenchymal water content from 3.7 to 3.9 mL g^−1^ (dry weight);
- over 5-7 min there was increased entry of both a fluorescent marker and an MRI marker, gadobutrol, both injected 15 min earlier into CSF in the cisterna magna; and
- over a similar period there was an increase in influx of Na^+^ and of mannitol radiotracers injected into CSF.

The main increase in influx of markers, starting at about t = 5 min (see Fig. 6a) occurred about 30 s after the appearance of a spreading depolarization [26] (see Sect. 3.2). Mestre et al. proposed that the spreading depolarization produced a strong vasoconstriction that *increased* the volume of the periarterial spaces allowing inflow of CSF. Such an inflow would produce a progressive increase in solute and water content of the parenchyma and this might account for the developing oedema. However, their results demonstrated only entry of Na^+^ from CSF in the earliest stages of ischaemic oedema reaching completion within 15 min (see Fig. 6). As noted by Mestre et al. elsewhere [116], the increase in water content and thus the intake of CSF, may be analogous to the CSF intake into the parenchyma that has been observed post-mortem [117, 118]. Such an intake does not represent a sustained increase in perivascular flow [2].

There are a number of observations which suggest that, with the exception of an initial transient as observed by Mestre et al. and just described above, perivascular transport is reduced, not increased, following ischaemia as listed below:

- Normal or increased perivascular transport would be expected to lead to much faster resolution of oedema fluid than is observed (see Sect. 7).
- Entry of DOTA-Gd into the parenchyma following cisternal injection is reduced after three hours of ischaemia implying reduced rather than increased influx via perivascular routes [119].
- The spreading depolarizations seen in another condition (migraine aura) are associated with decreased rather than increased perivascular transport [120].
- Once there is substantial astrocytic swelling, it is reasonable to expect that the swollen endfeet would reduce the size of the perivascular spaces [120, 121].

Further data are required to establish the extent and importance of CSF entry from subarachnoid spaces via perivascular routes in the subsequent development of oedema. In the following sections, it is assumed that most of the fluid that enters the parenchyma after the initial transient period does so via the blood–brain barrier.

#### Fluxes of solutes across the blood–brain barrier

Blood is another source of the NaCl that accumulates in oedema fluid. Unlike O_2_ and glucose, uptake of Na^+^ across the blood–brain barrier is slow and not blood-flow limited. Indeed, the rate of Na^+^ uptake required to account for development of oedema can be provided by even a severely compromised blood-flow, less than 1% of normal.^12^ Furthermore more than adequate uptake of NaCl from blood has been observed using radiotracers in MCAO in rats [122, 123].

Influx of Na^+^ and efflux of K^+^ across the blood–brain barrier are both favoured by the initial changes in ISF composition that take place following ischaemia (see Fig. 4). These changes affect both passive fluxes and active transport of these ions.

##### Effects on passive fluxes 

*Na* ^+^ *fluxes.*

 Under "normal" conditions, the passive fluxes of Na^+^ in both directions across the blood–brain barrier are much greater than the net flux. Initially, the likely mechanism for these passive fluxes is electrodiffusion via the paracellular spaces, see [124] and Sects. 4.3.4 and 4.3.5 in [4]. The decrease in [Na^+^]_isf_ in ischaemia is expected to reduce substantially passive efflux of Na^+^ from ISF to blood while leaving the passive influx from blood to ISF intact (compare [63, 125]). Thus, in ischaemia there will be a substantial net flux from blood to ISF which over four hours can amount to 120 µmol g^−1^.^13^ This represents a substantial fraction of the total uptake of Na^+^ into the parenchyma that has been observed following MCAO. 

*K*^+^ *fluxes.*

Under "normal conditions", the passive fluxes of K^+^ are expected to be much smaller than those of Na^+^ simply because the K^+^ concentrations in ISF and blood plasma are much smaller than those of Na^+^. In ischaemia, the more than tenfold increase in [K^+^]_isf_ derived from depolarized cells may lead to a substantial passive efflux of K^+^ from brain to blood.

##### Effects on active transport

**Fig. 7:**
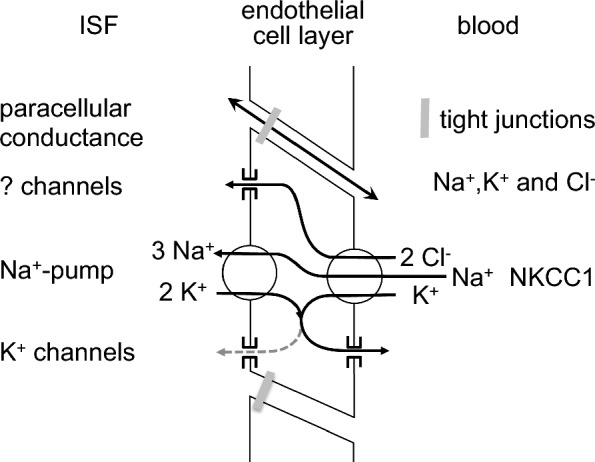
Suggested routes for Na^+^ and K^+^ transport across the endothelial cells of the blood–brain barrier in the early stages of ischaemic oedema. Passive fluxes of Na^+^, K^+^ and Cl^−^ probably occur via paracellular routes involving electrodiffusion that accounts for the endothelial conductance. Active transport through the cells is driven by the Na^+^-pump, a Na^+^, K^+^-ATPase: the increase in [K^+^]_isf_ stimulates this pump leading to Na^+^ flux from the endothelial cells into ISF and K^+^ flux from ISF into the cells. The resulting decrease in [Na^+^]_cell_ drives inward fluxes of Na^+^, K^+^ and Cl^−^ from blood into the cells via NKCC1. The net effect is transport of Na^+^ and Cl^−^ from blood to ISF and of K^+^ from ISF to blood. Many more transporters in addition to those shown are involved, prominently NHE1/2 as mentioned in the text, but the overall effect is as shown. There may be some recycling of K^+^ as indicated by the dotted line

**Fig. 8:**
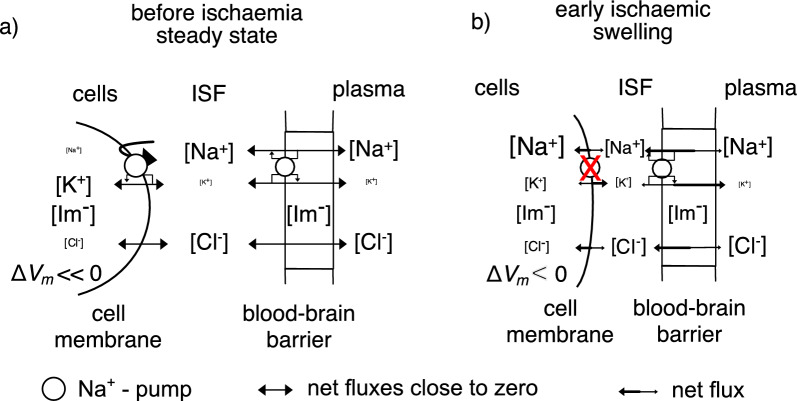
Diagram comparing movements of Na^+^, K^+^ and Cl^−^ into and out of parenchymal and endothelial cells before (left) and during (right) the initial phase of ischaemic swelling. Negatively charged impermeant solutes, Im^−^, in the parenchyma and in the endothelial cells provide part of the driving force for development of ischaemic oedema. Δ*V*_*m*_, is the cell membrane potential inside relative to 0 in plasma. **a** Before ischaemia, Na^+^ is effectively excluded from the cells in the parenchyma by their Na^+^- pumps. K^+^ is attracted into the cells and Cl^−^ repelled from them by the negative membrane potential, an example of the Donnan effect (see appendix E). The volumes of the cells and ISF are stable as are the ion concentrations, with the concentrations of Na^+^, K^+^ and Cl^−^ in ISF close to those in plasma. There may be a small net flux of solutes and water from plasma into ISF matched by a net flux out of the tissue into CSF primarily via perivascular routes (see [4]). **b** During the initial stages of ischaemia: the Na^+^-pumps are no longer able to exclude Na^+^ from the cells in the parenchyma but are still functional in the endothelial cells (see Sect. 3.5.2.2); Na^+^ and Cl^−^ enter parenchymal cells; K^+^ initially redistributes from neurons to astrocytes but eventually leaves both cell types; the cell membranes depolarize to small negative potentials; and the cells swell and ISF shrinks as described in Sect. 3.6. On a time scale of minutes to hours Na^+^ and Cl^−^ enter ISF across the blood–brain barrier at a rate that depends on the permeability of the barrier to these ions. Water follows down the resultant of the total osmotic and hydrostatic pressure gradients. The gradient for solute entry from the plasma persists because [Na^+^]_isf_ and [Cl^−^]_isf_ are kept somewhat less than the concentrations in plasma by continued entry of Na^+^ and Cl^−^ into the parenchymal cells. Development of oedema in the medium term, times from ~ 3 h to possibly 12 h (or more) is considered in Sects. 4 to 4.2.2 and Fig. 9

*Na*^+^-*pump activity and transcellular transport of Na*^+^*.* The Na^+^-pump on the abluminal side of the endothelial cells of the blood–brain barrier (see Fig. 7) may still function during focal ischaemia. Unlike the neurons and astrocytes, these endothelial cells are still exposed directly to O_2_ and glucose in the residual blood flow (compare [127]) and so can remain viable even in the face of extensive necrosis within the adjacent parenchyma (see Sect. 5). Their mitochondria thus still produce enough ATP to support active transport of Na^+^ by the Na^+^-pumps (see Fig. 8). Such continued activity of the pump encouraging Na^+^ entry into the parenchyma can be inferred from the results of Shigeno et al. [128] who found that ouabain, a pump inhibitor, was able to reduce oedema formation over 4 h presumably by preventing the Na^+^entry. Furthermore, as demonstrated by Schielke et al. [129] there is an increase in Na^+^ tracer influx during ischaemia. This would be expected if the Na^+^ pumps in the endothelium were still viable and were able to be stimulated by the raised [K^+^]_isf_ [127, 130–136].

The stimulated Na^+^-pump activity leading to reduction in [Na^+^] inside the endothelial cells will increase the gradient for Na^+^ entry from blood to cells (see Fig. 8b). Such entry [137] is likely to be via Na^+^, K^+^, 2Cl^−^ cotransport [138] (but see [139]) and Na^+^/H^+^ exchange [140–142]. There may also be some contribution from Na^+^, HCO_3_^−^ cotransport [140, 142, 143].

Entry of Na^+^ from blood replenishes the Na^+^ in the endothelial cells allowing further active transport into ISF by the Na^+^-pumps.

The importance of ion transport through the endothelial cells at the blood–brain barrier in the early development of oedema is supported by evidence from experiments using inhibitors of transporters known to be involved in Na^+^ and/or K^+^ fluxes into and out of the endothelial cells [137, 146]: These results show that formation of oedema over several hours after MCAO can be substantially reduced by:

- ouabain, an inhibitor of the Na^+^-pump [128];
- bumetanide, an inhibitor of NKCC1 [138];
- cariporide (HOE-647), an inhibitor of NHEs [147]; and
- TRAM-34, an inhibitor of KCa3.1 channels [148].

Furthermore, NKCC1 knock-out mice exhibited considerably less oedema and infarction than wildtype mice [149]. All of these results are as expected if a substantial proportion of the NaCl entry in the initial phase of tissue swelling occurs as a result of active transport across the endothelial cells.^14^

*Transcellular transport of K*^+^ Stimulation of the Na^+^-pumps by the increase in [K^+^]_isf_ will also affect K^+^ movements, in this case increasing K^+^ entry from ISF into the endothelial cells [127, 130–136] (see Sects. 6.3.4 and 6.6.4 in [4]). That raises their [K^+^] leading to increased K^+^ efflux into blood, possibly via ion channels [150–153](see Sect. 4.5.3 in [4]). This could plausibly account for the observed clearance of K^+^ from the parenchymal tissue across the blood–brain barrier under ischaemic conditions. K^+^ exit from the parenchyma partially offsets the increase in osmoles resulting from Na^+^ entry (see appendix D).

#### Production of osmotically active solutes within the parenchyma

The net influx of ions described above is not sufficient to maintain, let alone increase, the parenchymal osmolality during ischaemia. There must be extra osmoles derived from metabolism within the parenchyma.

Evidence for extra metabolites can be seen in MCAO experiments (see Fig. 5 and appendix D) but is more obvious in experiments on global ischaemia, i.e. where there is no blood-flow and hence neither a source of extra solutes from outside the parenchymal tissue nor washout of the extra metabolites. In these experiments it was found that the osmolality increased from 308 to 353 mOsmolal during 1 h of total ischaemia but there was little or no oedema [154]. When circulation was restored, so providing a source of water and further solutes, there was rapid brain swelling presumably driven by the already increased osmolality. Hossmann & Takagi [154] noted that the 45 mOsm increase substantially exceeded the ~ 20 mOsm expected from glycolysis and decomposition of labile compounds (see e.g. [155]) and concluded that there must be a release of osmoles from other brain constituents, e.g. by the catabolic break-down of proteins and lipids.

During focal ischaemia such as is produced by MCAO, the increase in osmolality due to extra solutes in the parenchyma (see column 7 of Table 1) is smaller than that seen in global ischaemia. This is partly because the solutes can be washed away in the residual blood flow and partly because they are diluted by influx of water.

In the initial stages of ischaemic oedema, the most obvious source of extra metabolites is the generation of lactic acid from glucose and glycogen. Some of that produced within the parenchymal cells is exported to ISF via monocarboxylic acid transporters (MCT). Both in cells and ISF, H^+^ will be buffered and thus not adding osmoles, but lactate^−^ will remain free and be osmotically active. The extra osmoles produced can amount to 15 µmol g^−1^ (per gram of wet tissue) or even 30–40 µmol g^−1^ with hyperglycaemia [155]. Hossmann [114] concluded that metabolically produced osmoles account for a substantial part of the increase in osmolality in the first hour or two following onset of ischaemia. Thereafter the increase in oedema is accounted for by a change in the amounts of Na^+^, Cl^−^ and K^+^. That in turn suggests that the metabolites are subsequently being washed away at a rate that balances their production but little is known about their rate of production during the medium and long term.

### Role of AQP4 in astrocyte endfeet

Deletion of AQP4 has profound effects on fluid movements in the brain. It has been shown to decrease the rate of net fluid transfer into the parenchyma during either water intoxication [162, 163] or the development of ischaemic oedema [162]. Furthermore, it has also been shown to decrease the rate of net fluid removal after either infusion of mock ISF or osmotherapy [164] and also decrease the rate of spread of fluid out of regions damaged by cold injury [165]. These effects are clear but because of the nature of the experiments, the actual flow rates via the AQP4-dependent routes could not be measured.

At the blood–brain barrier AQP4 is localized to the astrocyte endfoot membrane facing towards the vasculature. Thus, it is thought that a substantial portion of the water crossing the perivascular space to or from the blood can enter or leave via astrocyte endfeet.

AQP4 has also been linked to the effects of adrenergic receptor blockade on the development of oedema [166] and to the restoration of low [K^+^]_ISF_ after either cortical spreading depression or light-activated thrombosis [166, 167]. It has been proposed that both of these effects result from changes in AQP4 that modulate movements of fluid and K^+^ via glymphatics. While it is clear that AQP4 knock-out affects entry of solutes and presumably fluid via glymphatics [168, 169] it is not clear that this is via a direct effect on the water permeability of astrocyte endfeet ([170, 171]. For further discussion and references see [2].

It is interesting to note that there are marked changes in localization of AQP4 to endfeet in various stages of ischaemic oedema. Indeed it has been suggested this relocalization might be a useful target for therapy aimed at reducing the extent of oedema and of functional damage in ischaemic stroke and spinal cord injury [172, 173].

## The medium term: changes in the blood–brain barrier enhancing oedema formation

Events in the early stages have been considered in Sect. 3 above. These include discussion of the origins of the extra solutes and water in the parenchyma and the role of spreading depolarizations on expansion of the ischaemic core and oedema.

**Fig. 9:**
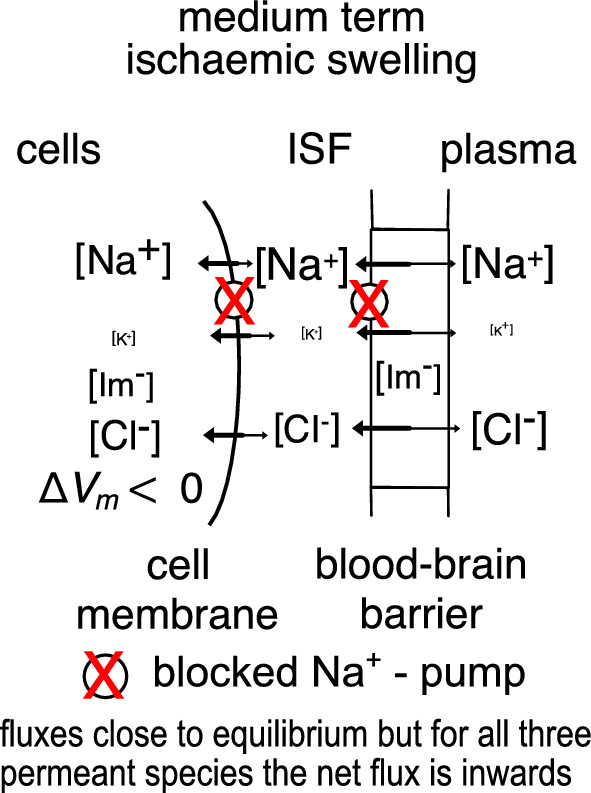
Diagram showing the movements of permeants, Na^+^, K^+^ and Cl^−^, into and out of parenchymal and endothelial cells in the medium term during ischaemic swelling. The Na^+^- pump is inhibited both in the parenchymal and endothelial cells (compare with situation in Fig. 8). The net negative charge on the impermeants (Im^−^) will lead to accumulation of Na^+^ and K^+^. If equilibrium could be reached (sufficient impermeants would need to be in plasma) their concentrations in the parenchyma cells would be slightly greater than in plasma. (For an introduction to more quantitative treatment see appendix E). However, during swelling entry of water keeps these concentrations slightly below those in plasma. There thus continue to be small gradients that drive influx of solutes like Na^+^ and Cl^−^ and this influx tends to increase solute concentrations which tend to increase the driving force for water entry. The net result is continually increasing amounts of solutes and water and thus tissue swelling

Starting 3 to 4 h after the onset of ischaemia oedema (see Fig. 9), changes in blood–brain barrier properties maintain the rate of oedema formation. The most important of these changes is increased permeability to NaCl. The medium term is here taken to start three to six hours and to finish perhaps nine to twelve hours after onset of ischaemia (see Fig. 1) though, as the approximate boundaries suggest, this is an arbitrary delineation since there is a continuum of changes in the processes occurring with the relative importance of different aspects shifting with time.

During the medium term, leakage of albumin into the parenchyma becomes apparent, clearly indicating that the permeability of the blood–brain barrier has changed.^15^ However, the leak is small and the albumin concentration in ISF remains substantially below that in plasma [174]. Furthermore, because movement of water across the blood–brain barrier is still governed by total osmotic pressure rather than colloid osmotic pressure, the critical barrier changes are to NaCl rather than to albumin. Hence as emphasized by Gotoh et al. [175] and Menzies et al. [176], the presence of albumin in the parenchyma does not explain the development of oedema in the first twelve hours. To explain this, at least two factors must be considered [18]: the maintenance of a driving force for uptake of NaCl and hence water into the parenchyma (see Sect. 4.1) and the increase in blood–brain barrier permeability to NaCl (see Sect. 4.2). Further production of osmotically active metabolites in the medium term appears not to have been investigated with the exception of the single study [114] described in Sect. 3.5.3).

### Maintenance of the driving forces for uptake of NaCl and water into the parenchyma

Once cells in the parenchyma have ceased to be able to pump Na^+^ (see Sects. 3.1 & 3.3) and have become permeable to Na^+^ (see below), their cell membranes are no longer an important barrier to movement of permeant ions and water into and out of the cells. In effect it is the impermeant intracellular molecules, e.g. proteins, nucleic acids, phosphate compounds, and the permeant ions that accumulate in response to the presence of the impermeants, that provide the osmotic driving force for water to enter (see Fig. 9 and appendix E).

### Increased permeability of the blood–brain barrier to NaCl

An important feature in the formation of oedema in the medium term is the increase in permeability of the blood–brain barrier to NaCl. This increase partly reflects changes in paracellular transport across the endothelial cell layer (see Sect. 4.2.2). However, on present evidence, another major, possibly more important change is the expression and opening of Na^+^-permeable SUR1-TRPM4 channels, both in the endothelial cells of the blood–brain barrier facilitating transcellular transport and in astrocyte endfeet aiding onward transport into astrocytes.

#### The involvement of SUR1-TRPM4 cation channels in the formation of oedema

SUR1-TRPM4 (also called NC_Ca-ATP_) is a non-selective cation channel involved in changes in blood–brain barrier permeability that lead to increased rates of oedema formation. SUR1-TRPM4 is formed by association of a SUR1 regulatory subunit with a TRPM4 channel. SUR1-TRPM4 is not expressed in uninjured brain and cannot be invoked to explain the earlier stage of oedema formation described in Sect. 3. However, during ischaemia there is transcriptional upregulation of both SUR1 and TRPM4 such that SUR1-TRPM4 becomes expressed in the plasma membranes of brain microvascular endothelial cells, astrocytes and neurons [173, 179–182]. Opening and closing of these channels depends on the level of ATP in the cells. ATP at normal levels binds to SUR1 and closes the associated TRPM4 ion channel, but at low ATP levels and in the presence of intracellular free Ca^2+^ the TRPM4 channel is open resulting in increased permeability of the blood–brain barrier to Na^+^ and K^+^ [18, 21, 173, 179, 183–186]. This, together with at least maintained permeability to Cl^−^, can plausibly account for at least part of the sustained oedema formation over the hours following the onset of ischaemia.

Part of the evidence that Na^+^ entry results from opening of SUR1-TRPM4 channels is the effectiveness of glibenclamide, (glyburide) an agent that binds to the SUR1 subunit and prevents opening of the associated SUR1-TRPM4.^16^ It has been found that glibenclamide substantially reduces oedema formation when given by continuous intravenous infusion starting immediately after the insult [179] or by loading dose plus infusion starting as late as 10 h after start of a 4.5 h period of ischaemia followed by reperfusion [183, 187]. If, as proposed (see [188] and [189] for review), glibenclamide extends the window during which reperfusion can be beneficial, this may turn out to be the most important feature of its use.

The ability of SUR1-TRPM4 to increase the rate of swelling implies that under these circumstances ATP levels are low. That in turn implies that the Na^+^-pump is unlikely to play a major role in Na^+^ transport. If not, the movements of Na^+^ across the blood–brain barrier must involve passive transport across both luminal and abluminal membranes as indicated in Fig. 9 [185]. The experimental result that inhibition of SUR1-TRPM4 substantially decreases fluxes and rate of swelling thus argues that a) with SUR1-TRPM4 channels open, a large proportion of the fluxes are transcellular and b) that in the medium to long term, active transport across endothelial cell membranes does not make a major contribution.

#### Paracellular: The impact of partial opening of tight junctions on oedema formation

Ischaemia leads to changes in the tightness of the blood–brain barrier. The endothelial cells of the blood–brain barrier are joined to each other by tight junctions which form a complete seal between the cells. Occludin and claudins are important molecular components of tight junctions that are found in epithelial layers throughout the body and also in certain endothelial layers. Differences in type and proportions of these components will determine the paracellular ionic permeability of any particular layer. Claudin-5 is the dominant claudin form in the endothelial lining at the blood–brain barrier and accounts for the much lower paracellular permeability of this interface compared to that of secretory epithelia such as the choroid plexuses [190, 191].

Changes in the properties of tight junctions following ischaemia are often described as occurring in two phases (see e.g. [192–195] but see also [196]). The first phase presumably begins soon after onset of ischaemia but may only become apparent after several hours. This manifests as an increase in permeability to small solutes (< 800 daltons) e.g. sucrose or a gadolinium probe. An increase in conductance of the blood–brain barrier, i.e. a decrease in transendothelial electrical resistance (TEER), may also be observed when opening is induced by addition of the chemokine CCL2 [197]. However, despite its importance, there has been no attempt to measure changes in paracellular permeability to Na^+^ and Cl^−^ during this phase. It is thought that the increase in permeability to small solutes may involve changes in conformation and position of claudin-5 [193, 197].

The second phase of barrier opening becomes apparent more than 12 h after onset of ischaemia and extends into days. It is associated with loss of tight junction structure and increase in permeability to a wider range of solutes than that seen during the first phase. This will be considered briefly in the next section.

## The long term: gross failure of the blood–brain barrier. Haemorrhagic transformation

The development of oedema after ~ 12 h will be discussed only briefly here. A good indication of the complexities of the cellular and molecular events occurring in the long-term in the parenchyma can be seen in other reviews [16, 23, 81, 198–205]. Also outside the remit of this review is any coverage of the changes that commit neurons to death by either necrosis or apoptosis including Ca^2+^ overload [50, 206–208].

As cell death becomes more prominent, solutes produced within the parenchyma by catabolism may produce large osmotic effects [209, 210]. As the oedema spreads carrying these solutes, their effects can be seen at some distance from their site of production even in regions where the blood–brain barrier remains intact [17].

The late phase of oedema is associated with severe loss of tight junction structure in the vasculature within the core leading to gross failure of the blood–brain barrier. There is extensive proteolysis of extracellular matrix and internalization of tight junction components into the endothelial cells from where they may be recycled or degraded [23, 197, 211, 212]). This accounts for the observed increases in permeability to a wide range of solutes. In some instances, the paracellular permeability of the blood–brain barrier to large and small solutes becomes sufficiently high that osmotic pressure gradients can be ignored and paracellular transport becomes effectively a hydrostatic pressure driven flow (compare [177, 178]). Indeed, the ultimate limitation on oedema development may be haemostasis as this removes the source of fluid.

In many instances focal ischaemia does not proceed to haemorrhage but in 10–15% of cases it does. This is called haemorrhagic transformation or conversion. Reperfusion using tissue plasminogen activator (tPA) after ~ 4.5 h (see Sect. 6) increases the risk of transformation. Factors important in transformation have been reviewed comprehensively by Jicking et al. [202] and Jin et al. [213]. The sequelae of transformation are similar to those when haemorrhage is the original fault [16].

Conversion of damaged tissue to final infarct (the region in which all cells have died) entails clearance of the cellular and extracellular debris, resolution of the oedema (see Sect. 7) and then growth of new cells including angiogenesis. Nervous tissue normally cannot be replaced and either the cellular component of the region of the infarct becomes a glial scar [214] [215–217]) or the volume ceases to be part of the parenchyma and is filled with CSF.

## Reperfusion

Immediate reperfusion may avoid damage but if it is delayed until the blood–brain barrier has been comprehensively breached or even destroyed then this will obviously lead to severe oedema at best and very likely to haemorrhage. At some stage, reperfusion shifts from being beneficial to being harmful.

It is not known how long a neuron can be exposed to ischaemic conditions before cell death becomes inevitable, partly because this varies with the type and location of the neuron and on the "severity" of the ischaemia. Some neurons are thought to be particularly vulnerable [29] which may account for the relatively short period, for example after cardiac arrest, that can be survived without life support. Most neurons located in the core in focal ischaemia are unlikely to be rescuable after a period of the order of an hour (see e.g. [29, 40, 41]). Those located in the penumbra, receiving somewhat higher blood flow, have a better survival chance but even in those regions where most cells will survive there can be selective neuronal loss over the same time period [218].

The principal objective of reperfusion is not to reverse changes within the core, but rather to prevent the core from spreading beyond its initial extent and to prevent as far as possible neuronal loss in the penumbra and surrounding regions. Within the core, reperfusion may be achieved but will have little benefit (and may be harmful). By contrast the sooner spreading of the core into the penumbra can be halted, the smaller the volume that will become part of the core (see for instance [218, 219]).

Reperfusion may be attempted by thrombolysis using alteplase (tissue plasminogen activator, tPA)) [220, 221] or, if the embolism is in an accessible artery, by thrombectomy [222]. According to the current National Clinical Guideline for Stroke for the United Kingdom and Ireland [223] up to 4.5 h after onset thrombolysis should be considered "regardless of age or stroke severity" (recommendation 3.5A). Between 4.5 h and 9 h thrombolysis should be considered if there is evidence "of the potential to salvage brain tissue" (recommendation 3.5B). The time window for thrombectomy is longer. When certain criteria are met thrombectomy should be considered even 24 h after the onset of stroke. The principal criteria are accessibility of the clot and evidence from imaging that there is still tissue that can be salvaged. Obviously, the sooner the better.

The main reason that the recommended time window for thrombolysis is shorter than for thrombectomy is that in addition to activation of plasmin and lysis of the clot responsible for the stroke, tPA has harmful effects promoting haemorrhagic transformation. Prominently it leads to secretion of matrix metalloprotease leading to lysis of extracellular matrix and breakdown of the blood–brain barrier [224].

## Resolution of oedema

There has been extensive work studying the resolution of oedema, particularly the oedema produced in the surrounding tissues either by focal freezing [225, 226] or by parenchymal infusions of fluids [227–232]. In these situations, it seems that during the time that fluid is accumulating either in the region of damage or at the site of infusion, the added fluid spreads through the surrounding tissue by pressure driven flow. Once the flow reaches white matter it tends to follow fibre tracts. If the fluid front reaches the brain parenchymal surface, it then flows into CSF [225, 228, 233]. However, in those cases where the oedema was produced by parenchymal infusion, this flow ceases shortly after the end of the infusion [234], or at least becomes much slower.

The excess volume introduced by mock CSF infusion in rats is removed from the parenchyma with a half-life of 12–24 h which is much faster than that after infusion of serum for which the half-life is several days [228]. This is most simply explained if there are two routes for removal of oedema fluid, transvascular and extravascular.

According to this explanation, mock CSF can be absorbed across the blood–brain barrier into the vasculature on a time scale of hours by a Starling-like mechanism. The Starling mechanism is too slow to have any significant impact on fluid movement across the blood–brain barrier under normal conditions (see Sect. 3.2.1 and appendix A in [1]) but, given many hours, slow passive absorption of fluid, limited primarily by the low permeability to NaCl, may account for the removal of colloid free fluid during resolution of oedema.

Serum cannot be reabsorbed effectively by the Starling mechanism because the proteins it contains provide an opposing colloid osmotic pressure [228]. Thus reabsorption after infusion of serum must occur by the slower extravascular route. A recent study on oedema following trauma suggests that this extravascular route is inhibited during traumatic oedema formation [235] which raises the possibility that extravascular resolution of oedema may also be inhibited. Further studies are certainly warranted.

## Overview and summary

Large scale oedema after trauma or stroke is life-threatening because it increases intracerebral pressure, *ICP* which may produce fatal brain herniation. However, it is, at present, not certain that small scale oedema is harmful; it may be a collateral effect of other events that do cause harm (appendix A).

Oedema is normally classified as osmotic, cytotoxic (e.g. ischaemic oedema), vasogenic (e.g. peritumoral oedema), periventricular (as seen in hydrocephalus (see [1]), and haemorrhagic or perihematomal (Sect. 1.1). The oedema associated with brain trauma is a mixture of cytotoxic, vasogenic and hemorrhagic oedemas (briefly mentioned in Sects. 1.1 and 5). Osmotic oedema occurs in water intoxication. Cytotoxic oedema arises from excess accumulation of solutes within cells. Vasogenic oedema arises from changes in the blood–brain barrier. This review considers primarily cytotoxic oedema arising from ischaemic stroke.

*Initial events* occurring during the onset of ischaemic oedema. These are described in Sect. 3. The blockage of blood vessels decreases blood flow and thus supply of O_2_ and glucose leading to rapid formation of a core region with irreversible damage and a surrounding penumbra in which still viable cells are at risk. The events occurring can be summarized as follows:

- Depolarization of neurons in the core, and neural uptake of Na^+^ and Cl^−^, loss of K^+^ and neuronal swelling (Sect. 3.1).
- Greatly increased [K^+^]_isf_ in the core triggers spreading depolarizations into the adjacent penumbral region. The metabolic cost of recovering from spreading depolarization without adequate blood supply is a major factor in the spread of the ischaemic core into the penumbra (Sect. 3.2).
- Astrocytic uptake of Na^+^, K^+^ and anions and swelling of astrocytes in the core (Sect. 3.3).
- Reduction in ISF volume by uptake into cells (Sects. 3.4).
- Increased solute content of the parenchyma by influx of solutes from CSF (Sect. 3.5.1) and blood (Sect. 3.5.2) and by production of additional solutes (Sect. 3.4 and appendix E) within the parenchyma (Sect. 3.5.3) leads to development of oedema (Sect. 3.5).
- Water enters primarily from blood across the endothelial layer with some passing directly into astrocyte endfeet via AQP4.

*Events in the medium term* (Sect. 4). These include changes in the blood–brain barrier that enhance oedema formation. There is a substantial increase in NaCl permeability of both parenchymal and endothelial cells. These changes include opening of SUR1-TPRM4 channels in neurons, astrocytes and endothelial cells, so allowing more rapid NaCl movements into and out of the cells (Sect. 4.2.1). In addition, there is an initial stage of opening of the tight junctions between the endothelial cells. In this initial stage, the important effect is an increase in permeability to small molecules (Sect. 4.2.2). The driving force for fluid entry into the parenchyma still arises from the presence of impermeant macromolecules in the parenchymal cells. Only substantially later does the blood–brain barrier become sufficiently leaky to large molecules, e.g. albumin, that their entry affects the driving force for fluid entry from blood.

Many aspects of tissue swelling in the medium term can be understood in terms of the Donnan effect caused by the excess of negative charge on large solutes trapped within the parenchymal cells. (Sects. 3 to 3.6 and 4.1 and appendix E).

*Events in the long term* (Sect. 5). At this point there is gross failure of the blood–brain barrier that occurs on a time scale of days. In about 10% of clinical cases this leads to haemorrhage into the tissue (haemorrhagic transformation). Lack of haemorrhage in 90% of cases implies that haemostasis has occurred.

*Treatment* At present the only effective treatment for ischaemic oedema is reperfusion (Sect. 6). If achieved sufficiently early it can greatly reduce the spread of ischaemic damage and oedema, but it cannot rescue the core. The major risk of reperfusion is haemorrhage.

*Resolution of oedema* (Sect. 7) This is a slow process requiring days or even weeks. Fluids without colloid, e.g. CSF or mock CSF, can be reabsorbed across the blood–brain barrier over a few days, presumably by the Starling mechanism with the rate limited by the permeability of the barrier to NaCl. If colloid is present in the fluid, e.g. after an infusion of serum, fluid must be removed by extravascular means which are much slower.

## Conclusions

The development of focal ischaemic oedema depends on events that occur in a localized region of the brain. Nevertheless, when the ischaemic region is sufficiently large, the oedema can produce marked increases in *ICP* with devastating results, i.e. herniation of brain structures accompanied by mechanical damage. By contrast, when ischaemia affects smaller regions, it is still uncertain that the oedema itself is responsible for any of the adverse consequences.

The early stages of ischaemic oedema development involve a complex interplay of ionic movements between neurons, astrocytes, endothelial cells, ISF, CSF and blood. Many of the main players have now been identified making it possible to explain how the oedema develops. Oedema fluid accumulates inside cells for two reasons: because of the intracellular generation of new, osmotically active solutes by catabolism and because of the Donnan effect of the negatively-charged impermeant intracellular solutes such as proteins and phosphate compounds. Once the ischaemia deprives the cells of the energy needed to transport Na^+^ outwards, the presence of these impermeants and the negative potential they produce lead to intracellular accumulation of Na^+^ together with Cl^−^ to maintain electroneutrality. The new metabolic products, the impermeants and the accumulated permeants provide the osmolality necessary for water to be retained in the cells. While there is coupled transport of solutes and water across the cell membranes, present evidence suggests that this is not sufficient in the face of the membrane permeability to water to produce measurable osmotic gradients between the cells and ISF.

When *ICP* is increased by large scale oedema, herniation of brain structures can be avoided by craniectomy or by aspiration of the osmotically active infarct region. However, neither of these procedures is reported to be effective in restricting the size of the damaged parenchymal region. The only satisfactory treatment to maintain or restore function following ischaemic stroke is restoration of blood flow providing this can be achieved relatively quickly. In the core region where there is little blood flow, the damage is irreversible, but in the surrounding penumbral region where there is somewhat higher blood flow, some functional recovery can be achieved even after a number of hours. One important objective of current research is to find treatments which increase the time window for successful reperfusion.

While the development of ischaemic oedema can be explained using plausible arguments, there are still questions that need to be answered. These include:

- Which transporters or channels allow entry of Na^+^, K^+^, HCO_3_^−^ and Cl^−^ (the permeants) into astrocytes? Measurements of concentrations of these ions inside astrocytes would be very useful.
- Do the differences between the isoforms of Na^+^-pump present in astrocytes and neurons noticeably affect oedema development?
- What is the importance of the localization of aquaporins to astrocyte endfeet? Can aquaporins be exploited to delay the development of oedema?

## Figures and Tables

**Fig. 10:**
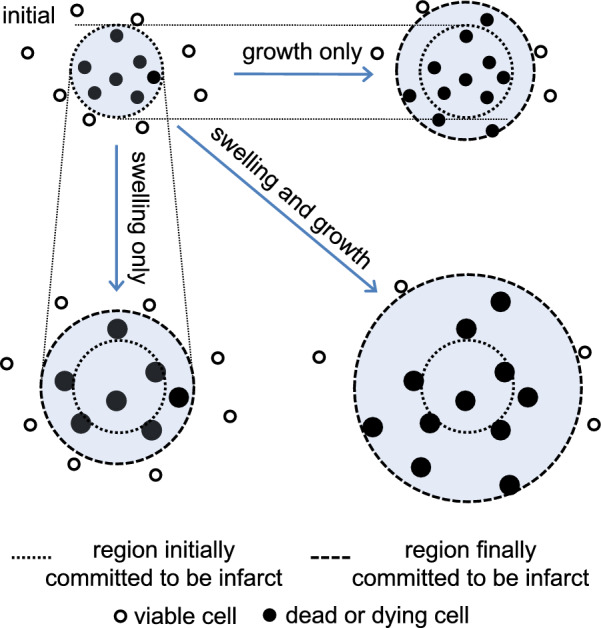
Schematic diagram showing possible ways in which growth and swelling of the core region can occur over time. Examples of cells present in and around the core are indicated by the small circles, (viable cells in white, dead or dying cells in black). The larger dotted circles indicate the size of the core shortly after the onset of ischaemia. The dashed circles represent the size of infarct after 2–3 days: upper right if cells outside the core region become incorporated but with no change in cell volume; lower left the size of an infarct if cells originally within the core swell but no extra cells from outside the original core are incorporated: lower right if both of these indicated changes take place

**Fig. 11:**
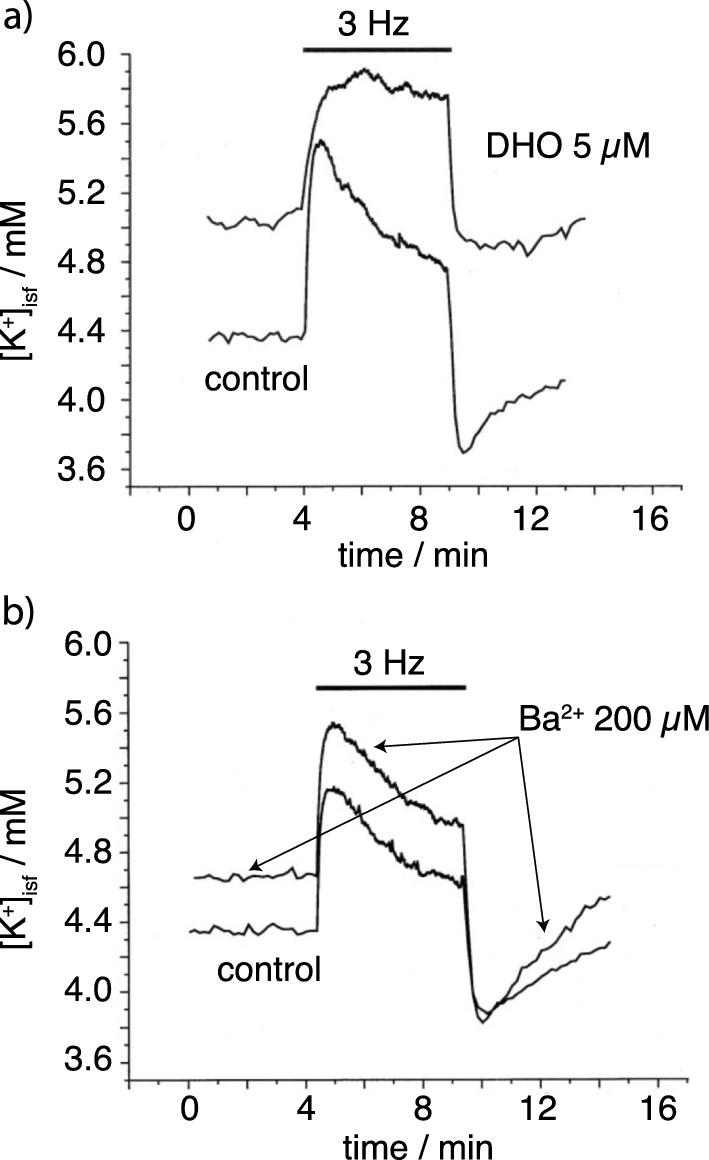
Effects of **a** dihydro-ouabain (DHO) and b) Ba^2+^ on [K^+^]_isf_. Concentrations were measured using ion-selective microelectrodes under baseline conditions and during antidromic Schaffer collateral 3 Hz stimulation of cells in the CA3 stratum pyramidale of rat hippocampus. Neuron firing releases K^+^ into the ISF. DHO is an inhibitor of Na^+^-pumps; Ba^2+^ inhibits Kir4.1 K^+^ channels known to be expressed in the astrocytes. Data traces are from D’Ambrosio et al. [265]. Each trace shows the baseline values before 3 Hz stimulation begins at 4 min. During this initial period, the rates of K^+^ release and uptake are in balance for the neurons and the astrocytes. In the control traces in both a) and b) at the onset of stimulation [K^+^]_isf_ increases rapidly to a peak then falls gradually as increases in [Na^+^]_neuron_ stimulate the neural Na^+^-pumps and thus increase the rate of reuptake of K^+^ into the neurons. At the end of stimulation, [K^+^]_isf_ decreases rapidly, overshoots the baseline and then increases towards baseline as [Na^+^]_neuron_ and the rate of K^+^ uptake into the neurons return to baseline values [265]. Both DHO in **a** and Ba^2+^ in **b** increase the baseline prestimulation [K^+^]_isf_ compared to its paired control suggesting that each inhibits one or more routes of removal of K^+^ from ISF. **a** With DHO present, at the onset of stimulation [K^+^]_isf_ increases rapidly to a higher and sustained plateau indicating that the rate of K^+^ removal from ISF is the same as the increased rate at which it is being released from the neurons. At the end of stimulation [K^+^]_isf_ decreases rapidly but with little overshoot. The absence of the gradual decline during stimulation and the lack of overshoot afterwards suggest that 5 µM DHO has completely inhibited the Na^+^ pumps in the neurons. This is consistent with the idea that the isoform of the pump in the neurons has a relatively high affinity for DHO [267]. In this interpretation, the release of K^+^ during stimulation would be balanced by uptake into astrocytes. From the evidence discussed so far this uptake could be via either a pump isoform with relatively low affinity for DHO or K^+^ channels. **b** With Ba^2+^ present, [K^+^]_isf_ is higher than in the paired control both before and during stimulation suggesting that Ba^2+^ has inhibited removal of K^+^ from ISF into the cells. At the end of stimulation, Ba^2+^ accentuates the size of the overshoot in [K^+^]_isf_ below the baseline before stimulation. This phenomenon is explained [265] by the combination of continued rapid uptake of K^+^ by the neural Na^+^-pumps stimulated by increased [Na^+^]_neuron_ together with inhibition by Ba^2+^ of the return of K^+^ to ISF from the astrocytes

## Data Availability

No new and unpublished data are reported in this review. There is no data to share.

## Appendix A

### Does oedema per se cause neuronal damage in ischaemia?

As discussed earlier, there is no doubt that severe wide-spread oedema is itself life threatening as it can increase intracranial pressure leading to brain herniation. The question here is whether less severe, more localized oedema can of itself cause damage to parenchymal cells or whether the damage which does occur is more directly the result of the ischaemia and spreading depolarizations.

In early work it was proposed that developing oedema was a key factor in spreading tissue damage. The tissue swelling was thought to compress blood vessels, perhaps just microvessels, reducing local blood flow and rendering surrounding tissue more ischaemic (see e.g. [236, 237]. The idea that there was compression of microvessels was based on the observation that much of the swelling during development of ischaemic oedema was in the astrocyte end feet surrounding microvessels [238, 239]. This view was never explicitly contradicted, but there was also little direct evidence for further reduction in blood flow as the oedema developed. The idea fell out of favour when it became apparent that spreading depolarizations account for at least part of the observed increase in core volume into the surrounding tissue (see e.g. [73, 240]. Takano et al. [48] showed it was the spreading depolarization increasing metabolic demand rather than any further deficit in blood flow that was challenging cell viability (see also [60]).

However, there is evidence that oedema per se has a role in neuronal damage. The size of an infarct is clearly a major determinant of the loss of neurological function. The "infarct" is the more or less sharply defined region in which all the cells are dead. There are two different but not mutually exclusive ways in which the volume of a region committed to becoming infarct can increase (see Fig. 10): by inclusion of more cells and structures or by swelling of the cells and structures already present within the region. Battey et al. [241] found in acute stroke subjects that the functional deficit measured on the modified Rankin scale 90 days after stroke correlated with the final size of the infarct but also and independently with the extent to which oedema contributed to the infarct expansion. Oedema even of extent as small as 11 mL worsened the outcome. In other words, given two infarcts of similar size, functional outcome was worse in the patient with greater oedema. Since all function of neurons within either of these two infarcts is irretrievably lost, regardless of the extent of oedema, any differences in neuronal function to be ascribed to effects of oedema must relate to effects outside the infarct. The suggestion is that oedema somehow causes an extra impairment or loss of function in tissue adjacent to the infarct, presumably from selective neuronal loss [218].

Further evidence for a role for oedema per se in neural damage has been obtained using glibenclamide and MCAO in rats. In a 4.5 h transient MCAO model, it was found that oedema, behavioural deficit and mortality determined at 28 h after occlusion could all be reduced by treatment with glibenclamide starting 4.5 h or even 10 h after occlusion. By contrast the final infarct volume was not reduced [187], see also [242]. The ability of glibenclamide given after 4.5 h to reduce behavioural deficit but not infarct size indicates that glibenclamide is somehow involved in limiting neural damage additional to that occurring within the infarct. Furthermore, because glibenclamide also reduces oedema, it is possible that the effect on behaviour results from its effect on oedema.

Further evidence that oedema produces adverse effects was reported by Woo et al. [186] who found that the antisense oligodeoxynucleotides they used to knock-down SUR1-TRPM4 significantly reduced oedema (calculated from stained sections as in [243])^17^ and loss of function assessed as spontaneous movement but had no effect on infarct volume. All of these were measured 24 h after occlusion. Similarly Stokum et al. [173] found in mice that, when results after MCAO with different reperfusion times were compared, there were worse functional outcomes when oedema was greater for the same final infarct size.

Whether targeting the formation of oedema with the aim of increasing retention of function will ultimately lead to useful treatment of ischaemic stroke is still not known. Stokum et al. [21] and Robert et al. [244] have presented interesting overviews of the various treatments being considered in 2020.

## Appendix B

### Lactic acidosis in ischaemia

The term acidosis refers to the processes leading to a reduction in the pH of a body fluid. “Lactic acidosis” thus means that pH is reduced as a result of lactic acid production. There has been considerable controversy about the use of this term [245–250] because the reactions that produce lactate^−^ from glucose do not in themselves produce the protons needed for lactic acid [155, 251]. Thus writing the reactions in a way that displays conservation of charge

1 $$ {\text{glucose }} + {\text{ 2 Pi}}^{{{2} - }} + {\text{ 2 ADP}}^{{{3} - }} + {\text{ 2 NAD}}^{ + } {-\!\!-} > {\text{ 2 pyruvate}}^{ - } + {\text{ 2 ATP}}^{{{4} - }} + {\text{2 NADH }} + {\text{ 2 H}}^{ + } + {\text{ 2 H}}_{{2}} {\text{O}} $$

and

2 $$ {\text{2 pyruvate}}^{ - } + {\text{2 NADH }} + {\text{ 2 H}}^{ + } {-\!\!-} > {\text{ 2 lactate}}^{ - } + {\text{2 NAD}}^{ + } $$

which together become

3 $$ {\text{glucose }} + {\text{ 2 Pi}}^{{{2} - }} + {\text{ 2 ADP}}^{{{3} - }} {-\!\!-} > {\text{ 2 lactate}}^{ - } + {\text{ 2 ATP}}^{{{4} - }} + {\text{ 2 H}}_{{2}} {\text{O}}. $$

It is clear that these reactions do not result in a net production of any acid, i.e. H^+^. In that case how can they be the origin of acidosis?

However these reactions should not be considered in isolation. They are occurring to regenerate the ATP that is being consumed in performing the work being done by the cell. When the ATP consuming reactions,

4 $$ {\text{2ATP}}^{{{4} - }} + {\text{ 2H}}_{{2}} {\text{O}}{-\!\!-} > {\text{ 2Pi}}^{{{2} - }} + {\text{ 2ADP}}^{{{3} - }} + {\text{ 2H}}^{ + } + {\text{ heat and energy to perform work}}, $$

are combined with anaerobic glycolysis, the overall reaction supporting the work of the cell is

5 $$ {\text{glucose }}{-\!\!-} > {\text{ 2lactate}}^{ - } + {\text{ 2H}}^{ + } + {\text{ work }}\& {\text{ heat}}, $$

i.e. the anaerobic metabolism of glucose to obtain the energy needed for work does produce lactic acid and is responsible for a fall in pH. Thus, to the extent that these are the reactions occurring, calling the acidosis “lactic acidosis” is appropriate [155]. The role of production of lactic acid in normal pH regulation in the CNS was reviewed in Sect. 6 of [4].

The controversy over the name “lactic acidosis” isn’t entirely a matter of semantics: there is a substantive issue concerned with the acidosis observed with ischaemia. In this situation, part of the production of acid results from the breakdown of ATP and other forms of high energy phosphate compounds already present within the cell, hence to the extent that the acidosis results from such breakdown it has nothing to do with lactate. However, because substantially more lactate can be produced than existing ATP + creatinine phosphate (see e.g. [102]) much of the reduction of pH inside the cell depends on lactate production. Thus the term lactic acidosis (or lactacidosis), which has been used for many years (see e.g. [155, 253, 254]), is still appropriate.

## Appendix C

### Ischaemic responses of neurons and astrocytes

Na^+^-pumps are key players: (a) in the long-term regulation of intracellular and extracellular Na^+^ and K^+^ concentrations, (b) in the reuptake of K^+^ and expulsion of Na^+^ after action potentials in nerve; and (c) in the redistributions of Na^+^ and K^+^ in the cells and interstitial fluid occurring in ischaemia. For the first of these roles the important pumps are located in the endothelial cells of the blood–brain barrier while for the second they are located in neurons. Pumps in neurons, astrocytes and endothelial cells are important during the development of oedema until the ATP/ADP ratio drops sufficiently to stop their function.

### Neurons

#### Ions

There are likely to be at least four types of ion channels that can allow the early Na^+^ entry into neurons in ischaemia including voltage excitable Na^+^ channels and NMDA cation channels that are opened by glutamate^−^. It is possible to inhibit the Na^+^ entry and depolarization with a cocktail of cation channel inhibitors (TTX which block voltage excitable Na^+^ channels, CPP which blocks NMDA cation channels, DNQX which blocks other glutamate^−^ activated channels, and Ni^2+^ which blocks various cation channels) [255], but, by omitting any one of these, spreading depolarizations (see below) recorded from CA1 pyramidal neurones in rat hippocampal brain slices [43] (see also [256]) could still occur. (The multiplicity of channels and overlap in their functions may be part of the explanation for why it is difficult to achieve beneficial clinical effects in stroke with inhibitors of any single type of channel.) Ion channels and transporters involved in oedema are reviewed in [257].

In the normal resting state of a neuron there is a small inward leak of Na^+^ balanced by an outward flux via the Na^+^-pump. The resulting inward flux of K^+^ via the pump and an outward flux of Cl^−^ by secondary active transport via a K^+^, Cl^−^ cotransporter, probably KCC2 [46], are in turn balanced by fluxes in the opposite directions, presumably via channels. The pump-leak balance maintains resting [K^+^]_neuron_ above and resting [Cl^−^]_neuron_ below equilibrium with the membrane potential such that increasing the passive permeability to either ion produces hyperpolarization (e.g. an inhibitory postsynaptic potential). In the ischaemic core the inhibition of the Na^+^-pump when ATP is depleted leads to depolarization, caused primarily by the then unopposed Na^+^ entry. The depolarization leads to an increase in K^+^ permeability and release of K^+^ which decreases [K^+^]_neuron_ and increases [K^+^]_isf_. The change in the membrane potential also increases Cl^−^ transport into the neurons. Because the cell contents must remain almost neutral, the gain of Na^+^ must be close to the sum of the loss of K^+^ and gain of Cl^−^. Any departure from electroneutrality large enough to be detected by means other than by measuring the potential would produce an impossibly large electrical potential. (There is no need to invoke specific binding of K^+^ (as in [18]) to explain why the gain of Na^+^ exceeds the loss of K^+^ and there is no evidence for such binding.)

#### Water

The net gain of solutes in the neuron has frequently been stated to be the cause of neural swelling, tacitly assuming that the influx of water is osmotically driven. For this to be correct, the water permeability of the neuron must be adequate. The osmotic water permeability of pyramidal neurons with nearly normal resting potential in tissue slices is remarkably low. As Andrew et al. [49] note, this implies that these neurons do not have aquaporins and in addition that they are among the “cell types … that resist high osmotic gradients with specialized membranes that impede the equilibration of water between compartments”. Andrew et al. suggest that the rapid swelling observed during depolarization can be explained if some of the channels or transporters opened or activated by the depolarization are permeable to water.

Steffensen et al. [44] have suggested an alternative explanation for rapid water entry into dendritic beads during depolarization. They conclude that the water entry occurs by coupled transport through cotransporters like the Na^+^,K^+^,2Cl^−^ transporter NKCC1 and the lactic acid transporter MCT2. In support of their suggestion, they report that omission of Cl^−^ or use of inhibitors of NKCC1, (furosemide) and MCT2 (4-CIN) reduces swelling without affecting spreading depolarization. However, this evidence does not establish that cotransport of water is essential for swelling in dendritic beads or in cell bodies. For swelling to occur, there must be entry of cations, anions and water. All the available evidence can be explained if there is inhibition of anion entry reducing the osmotic gradient that would be needed to drive osmotic entry of water. In the dendritic beads, the inhibition of NKCC1 activity would thus inhibit swelling regardless of the route of water entry. (Neither Cl^−^ entry nor NKCC1 activity are needed for spreading depolarization.) In cell bodies, cell swelling was not inhibited by bumetanide (an inhibitor of NKCC1) within a few minutes of persistent activation of Na^+^ channels but was blocked by DIDS and siRNA directed against the Cl^−^ transporter slc26a11 [45], which is also consistent with the primary importance of the inhibition of anion transport. The two hypotheses for the mechanism of swelling could be distinguished if the osmotic water permeability of the depolarized neurons were known, but apparently those experiments have not yet been done.

### Astrocytes

#### Ions

Based on evidence obtained with astrocytes in cell culture it has been proposed that most of the K^+^ and Cl^−^ entry into astrocytes is mediated by the Na^+^, K^+^, 2Cl^−^-cotransporter, NKCC1 [258–260]. However, while NKCC1 expression in cultured astrocytes is high, that in astrocytes in vivo is low, reported as being either undetectable [261] or comparable to the low levels seen in adult neurons [262] (for extensive discussion see [96] but for a contrary view see [90]). Consistent with low expression of NKCC1, furosemide, an inhibitor of NKCC1, had negligible effect on the change in [K^+^]_isf_ when K^+^ was added iontophoretically [263].

MacAulay [96] has extensively reviewed studies on the changes in [K^+^]_isf_ and the volume of the extracellular space following repetitive nerve stimulation. Some care is required in comparing those results with the changes in ischaemia. Following nerve stimulation there is reuptake of K^+^ into neurons as their Na^+^-pumps are stimulated by the increase in [Na^+^]_neuron_ (see e.g. [264, 265] and Fig. B-1) but with the fall in [ATP] in ischaemia this reuptake will be much less obvious. Similarly, because ATP becomes depleted in ischaemia, the role of the Na^+^-pumps in astrocytes is likely to differ between the response to nerve stimulation and that to ischaemia. Nevertheless, results obtained during and after repetitive nerve stimulation (see Fig. B-1) do still inform any discussion of the mechanisms involved in responses to ischaemia.

The account in [96] assigns only a restricted role to the K^+^ conductance of astrocytes in the response to nerve stimulation, noting that it sets the resting potential and thus is important in the initial astrocytic depolarization when [K^+^]_isf_ is increased. The uptake of K^+^ into astrocytes is said to occur primarily via their Na^+^-pumps [86, 95, 96, 265]. However, while the importance of Na^+^-pumps in reuptake of K^+^ into neurons is well established, the relative importance of astrocytic Na^+^ pumps and K^+^ channels in K^+^ entry into astrocytes is not so clear. Data shown in Fig. 11 from the meticulous study by D'Ambrosio et al. [265] provide a good example. The interpretation in the legend to Fig. 11 differs from the interpretation given by D'Ambrosio in that it takes into account the strong possibility that neurons and astrocytes have different isoforms of the Na^+^-pumps with different affinities for inhibitors [266–269]. Reinterpreted, D'Ambrosio et al.'s data suggest a role for Ba^2+^-sensitive K^+^-channels in entry of K^+^ into astrocytes both during and after stimulation of K^+^ release from neurons.

It is unfortunate that the study by D'Ambrosio et al. [265] did not report data either for a sufficient concentration of the Na^+^-pump inhibitor to inhibit the pumps in astrocytes or for the combination of pump inhibitor and Ba^2+^. These results might have allowed a clearer distinction between pump-mediated and channel-mediated fluxes of K^+^ into astrocytes.

In the account given in Sect. 3.3, K^+^ conductance is given more prominence (compare [270]) than in [96] for two principal reasons, first in ischaemia the Na^+^-pumps in the astrocytes are to some extent inhibited and secondly some route for inward current is required to maintain approximate electroneutrality during astrocyte swelling (see Fig. 4). In the initial changes during ischaemia, both NBCe1 and the Na^+^-pump, if functioning, are expected to carry outward currents, NBCe1 as a net inward movement of negative charge, 2 HCO_3_^−^ for each Na^+^, and the Na^+^-pump as an outward movement of positive charge, 3 Na^+^ outward for each 2 K^+^ inward.

The only plausible routes for an inward current are K^+^ entry via the K^+^ conductance and Na^+^ entry via charge carrying cotransporters with Na^+^- glutamate cotransport being the most prominent [271].

The stoichiometry of the Na^+^, glutamate^−^-transporter is complex, but it mediates a net current into the astrocyte, which will tend to depolarize it, while at the same time being a source of intracellular anions. Thus, it might be expected to account for some of the volume increase in astrocytes and the ISF decrease. However, inhibition of coupled glutamate transport with the inhibitor TBOA (200 µM, DL-threo-b-Benzyloxyaspartic acid) produced a modest potentiation of the nerve-stimulus-induced decrease in ISF volume, i.e. a change in the reverse direction of that expected if the astrocyte volume increase and ISF volume decrease were the results of coupled transport of glutamate into the astrocytes [95]. Hence, by a process of elimination, that leaves K^+^ entry as the likely basis for the inward current.

There are a number of arguments in favour of channels mediating K^+^ entry into astrocytes in ischaemia including:

- K^+^ channels are present and at least initially open;
- [K^+^]_isf_ is greatly increased while [Na^+^]_isf_ is substantially reduced, favouring importance of K^+^ entry mechanisms;
- Ba^2+^, a Kir channel blocker, has been found to block most of the K^+^ entry into glia [69] (but see caveat below);
- Ba^2+^ augments the change in [K^+^]_isf_ when K^+^ is added iontophoretically [263];
- deletion of the Kir4.1 subunit in mice abolished buffering of [K.^+^]_isf_ in the ventral respiratory group [272]
- both baseline and nerve simulation increased [K^+^]_isf_ are higher in the presence of Ba^2+^ (see Fig. 11 and discussion above).

It would be very interesting indeed to know how [K^+^]_astrocyte_, [Na^+^]_astrocyte_, [Cl^−^]_astrocyte_, [HCO_3_^−^]_astrocyte_ and *pH*_astrocyte_ vary in intact tissue during ischaemia both initially when [K^+^]_isf_ is first increased and also subsequently. However, experiments reporting changes actually recorded within astrocytes, as opposed to being inferred from those recorded in ISF, have been rare. Ballanyi et al. [69] used sharp microelectrodes to penetrate glial cells. Unfortunately, there is uncertainty about whether the glial cells in their experiments were astrocytes or oligodendrocytes [85] (or NG2 cells [270]) and thus further investigation is required (compare [65]). Du et al. [273] used patch clamp recording in slices from control mice and those with knock-out of either TWIK-1 or TWIK-1 and TREK-1 K^+^ channels and found no difference in their passive conductance or input resistance, suggesting that some other type of channel (the obvious candidate being Kir4.1) is responsible for the resting conductance of astrocytes. By contrast Junsung Woo et al. [89] loaded individual cells with a K^+^ sensitive dye via a patch pipette and recorded transients in neurons and astrocytes in response to neural stimulation. Not surprisingly they found that K^+^ was released from the neurons and taken up by the astrocytes. However, they found that the uptake in astrocytes was blocked by shRNA knockdown of TREK-1 channels and not by knockdown of Kir4.1. Which K^+^ channels are most important is not yet certain and may not be the same in all astrocytes [89, 273–276].

There is likely to be some recycling of HCO_3_^−^ and some entry of Cl^−^, but the mechanisms for these are unknown. One suggestion has been HCO_3_^−^ / Cl^−^ exchange [277] [278] [279, 280] but the efflux of HCO_3_^−^ from astrocytes after repetitive nerve simulation appears to be by reversal of NBCe1 rather than HCO_3_^−^ / Cl^−^ exchange [281].

#### Water

MacAulay [96] describes the mechanism for water uptake from ISF into astrocytes as being primarily cotransport of water along with Na^+^ and HCO_3_^−^ by NBCe1. (There is also an incompletely defined role for monocarboxylate transporters, MCTs). Much of the evidence in favour of this proposal is that inhibition of NBCe1 reduces astrocyte swelling [95, 96]. However, to the extent that anion entry into astrocytes requires NBCe1, its inhibition would tend to hyperpolarize the cells and inhibit their swelling, regardless of whether the net entry of water was occurring by cotransport or via osmotically driven fluxes.

The argument against water entry by osmotically driven water fluxes given by MacAulay is based on their result [96, 282] (see also [283]) that inhibition of AQP4 does not reduce the rate of astrocyte swelling and ISF volume reduction following repetitive nerve stimulation. Set against that, however, are conflicting results showing that knock-out of AQP4 reduces [162] or knock-down virtually abolishes [89]) the swelling response. Furthermore, if inhibition of AQP4 does fail to affect the response that would only argue against a requirement for AQP4 not against osmotically driven water movement through other routes including the lipid portions of the membrane. As noted in [96], Solenov et al. [284] found that at 37 ºC the water permeability of cultured astrocytes from AQP4 knockout mice was still about half that of those cultured from wild-type mice which might be sufficient to allow rapid water entry. Further study is required to clarify the roles of AQP4, of osmotic movements of water, and of co-transport of water via NBCe1.

## Appendix D

### The early changes in tissue volume, Na^+^ and K^+^ content and osmolality following onset of ischaemia.

These studies are now more than thirty years old. Despite this there does not seem to have been any compilation of the results. Nor has there been any discussion of the relative merits of the assertions that tissue swelling (water gain) is driven primarily by the metabolic production of new osmoles or that the swelling is due to accumulation of NaCl. The conclusion reported in Fig. 5 based on consideration of the data in Table 1 is that both are important. Three reports were excluded from calculations of the fraction of swelling attributable to NaCl entry, because in two cases [285, 286] data from at least 3 h of ischaemia were not included and in one case [112] changes in Na^+^ and K^+^ were not reported.

The mean values of the quantities calculated from the experiments in Table 1 (with the three exclusions discussed above) are: water gain, 0.83 mL g^−1^ (scaled to 4 h time point); ionic osmolality in the added fluid (at the time points of the data), 177 mOsmolal; osmolality attributable to new osmoles produced within the tissue, 133 mOsmolal. Based on these values the water gain over 4 h attributable to uptake of NaCl and loss of KCl is 0.47 mL g^−1^ and to production of new osmoles 0.36 mL g^−1^. Given the scatter of the data, these are only rough estimates.

One of the reasons why the data are scattered may be the different ways in which the parenchymal samples were obtained for measurement of water and solute gains. These varied from using an entire hemisphere subjected to MCAO [286, 287] to using small cubes, 1 mm^3^, dissected from the MCAO core [176, 288, 289]. It is difficult to say how these differences may have affected the results reported in Table 1 because with the exceptions of [176] they are not discussed in the original papers (see also [289]). However, the errors introduced by using the whole hemisphere are probably not as great as might be imagined. To see this, consider a simple, artificial example of the calculations of fluid uptakes per unit volume when all of the uptake into a hemisphere with volume *V*_hemi_ occurs within a portion with volume *V*_sample._. Because the estimate of the uptake per unit volume is just the measured uptake divided by the volume, the ratio of the estimates obtained by taking the hemisphere instead of the sample for analysis would be just the ratio, *V*_sample_/*V*_hemi_. Use of the hemisphere as the sample will lead to underestimation of the uptake per unit volume, but the errors may be little more than a factor of 2 when the oedematous region is large.

The osmotic effects of entry of NaCl from outside the parenchyma and the production of new osmoles within the parenchyma are much larger than any conceivable effects of influx of serum albumin from blood or of hydrostatic pressure differences between the tissue and blood. The largest conceivable osmolality attributable to influx of serum albumin from blood would be a few mOsmolal. The largest conceivable hydrostatic pressure difference would be less than 19 mmHg which as a driving force for fluid movement is equivalent to 1 mOsmolal. Thus influx of colloid and hydrostatic pressure gradients almost certainly do not drive the early development of ischaemic oedema (compare [174, 175, 285]).

**Table 1:** The early changes in volume and fluid composition in regions of brain parenchyma subject to ischaemia following middle cerebral artery occlusion (MCAo) or carotid artery occlusion in the case of gerbil studies

Species	Time after insult/h	water gain/mL g^−1 a^	Na^+^ gain/µmol g^−1 b^	K^+^ loss/µmol g^−1 b^	ionic osmolality of added fluid/mOsmolal ^c^	Δosmolality/mOsmolal ^d^	Ref. ^e^
cat	2	0.56	69	88	− 67		Bartko et al. [285]
gerbil	3	0.76	185	130	145		Ito et al. [287]
cat	4	0.7	192	137	157	16–22	Schuier and Hoss-mann [102]
cat	4	0.72				9	Hossmann [112]
rat	4	1.91	263	− 39	316		Young et al. [63]
gerbil	3	0.44	146	108	173		Lo et al. [125]
rat	4	0.4	139	141	− 10	18	Hatashita et al. [113]
cat	4	0.7	178	114	183^e^	9	Hossmann [114]^f, e^
gerbil	3	0.53	160	91	260		Betz et al. [288]
rat	2	0.55	228	95	483		Yang et al. [286]
rat	4	0.65	178	116	191		Menzies et al. [176]

^a^This column denotes the change in volume measured in terms of water content per unit dry weight of tissue, i.e. the solids after the water is evaporated. (For the calculations leading to Fig. 5, water gains measured after three hours were presumed to continue at the same rate for an additional hour.) The water gain at any time point can be calculated [290] from values for the percentage of water in the tissue (taken to be milliliters per 100 g of tissue wet weight). The water content per unit dry weight both before and after the period of ischaemia can be calculated as % water / (100-% water) and the gain is then the difference. In Fig. 5 the water content per unit dry weight before ischaemia is taken as 8 mL g^−1^

^b^These columns show the content of Na^+^ or K^+^ measured per unit dry weight of tissue

^c^In this column the ionic osmolality of the added fluid has been calculated from the previous columns assuming Cl^−^ gain = Na^+^ gain—K^+^ loss (net gain of Na^+^, K^+^ and Cl^−^) / (water gain) = 2 x (Na^+^ gain—K^+^ loss) / (water gain). The calculation of the fraction of the swelling that can be attributed to net uptake of NaCl minus loss of KCl shown in Fig. 5 and listed below is based on the assumption that the osmolality of ISF during swelling is 310 mOsmolal which is close to the sum of the initial osmolality, assumed to be 296 mOsmolal, plus the average of the few values of the increase in osmolality that have been determined experimentally. The fraction is then the ionic osmolality of the added fluid / 310 mOsmolal. The remainder of the osmolality is assumed to be provided by the production of new osmoles within the tissue

^d^Δosmolality is the total osmolality 2 or 3 h after onset of ischaemia (depending on the study) minus that before onset

^e^Murtha et al. [291] reported smaller, not significantly different from zero, oedemas measured 24 h after onset of 3 h transient MCAO. These data could not be used because they didn't report oedema at any earlier time points

^f^Hossmann discusses the sequence of increases in metabolically produced osmoles and influx of NaCl with the former occurring at the beginning and the latter proceeding with a delay of about an hour compared to the uptake of water. This suggests that subsequent increase in oedema is accompanied by ~ 310 mOsmoles of ions per litre gained less than 19 mmHg which as a driving force for fluid movement is equivalent to 1 mOsmolal. Thus influx of colloid and hydrostatic pressure gradients almost certainly do not drive the early development of ischaemic oedema (compare [175, 176, 288])

## Appendix E

### The Donnan effect, cell swelling and oedema

The electrical potential difference across the cell membrane separating the contents of a cell from its outside is equal to the net charge within the cell divided by the capacitance of the membrane. Thus, for cells with negative potentials, there is an excess of negative over positive charge within the cell. However, over the entire range of membrane potentials observed, the difference between the amounts of negative and positive charge present is very much smaller than either of those amounts, i.e. the amounts of negative and positive charge are almost equal. When determined in any way other than by measuring the membrane potential, the net charge is within experimental error of being zero: the cell contents appear to be neutral. The approximation used in calculations that the cell contents are neutral even though the potential difference is not zero is the so-called Principle of Electroneutrality (see e.g. Section 6.1.2. in [4]).

Cells contain large amounts of impermeant negatively charged solutes, e.g. proteins, nucleic acids, and phosphate compounds. Because otherwise membrane potentials would be prohibitively large, the permeant solutes must be distributed to balance that charge. In practice this means the cells must contain more K^+^ than Cl^−^. Given two important assumptions, (a) that solutes can be divided into those that are effectively impermeant and those that are permeant and (b) that water reaches osmotic equilibrium, the Donnan equilibrium describes quantitatively the relations between the amounts and charges of the impermeants, the concentrations and charges of the permeant ions, the membrane potential and cell volume.^18^ The Donnan effect is the tendency for the permeant ions and water to move so that the concentrations and volume approach this equilibrium. The Donnan potential is the membrane potential at Donnan equilibrium. The Donnan equilibrium [292, 293] was introduced into cell physiology by Boyle and Conway [294] who used it to account for the cell volume changes and distribution of ions observed in frog skeletal muscle fibres.

To better understand the consequences of the Donnan effect, consider what would happen if a cell like a muscle fibre were suspended in just KCl. Because the negative potential would lead to more accumulation of K^+^ than depletion of Cl^−^, [K^+^]_inside_ + [Cl^−^]_inside_ would be larger than [K^+^]_outside_ + [Cl^−^]_outside_ and the osmotic pressure inside the cell would be higher than that outside which would drive cell swelling.^19^ For the fibre to have a stable volume either the osmotic pressure must be balanced by a higher hydrostatic pressure acting on the fluid inside the fibre than on the fluid outside or there must be some impermeant solute that can increase the osmotic pressure of the solution outside. For mammalian cells with membranes that cannot support significant hydrostatic pressure differences, this means in practice that there must be external impermeants. An important role of the Na^+^-pump is to render the cell membrane effectively impermeant to Na^+^ so that the Na^+^ inside acts as one of the internal impermeants and the Na^+^ outside, present at a relatively high concentration, can act as the external impermeant that allows osmotic balance. For muscle fibres Boyle and Conway showed that the experimentally measured distributions of K^+^ (high inside) and Cl^−^ (low inside), the membrane potential (negative inside) and the cell volume were interrelated as predicted by Donnan’s equations. The initial swelling of brain cells surrounded by ISF in the early stages of ischaemia has been described in Sects. 3.1–3.3, Fig. 4, Fig. 8 and appendix C in terms like those used by Boyle and Conway.

The Donnan theory can also be used to interpret data for a gel with a flexible framework of negatively charged molecular strands with the spaces between them filled with solution. Such a gel can expand and shrink taking up and releasing fluid and ions. To be able to resist expansion, the gel strands must be cross linked. Stable volumes can then be achieved because stretching of the tissue matrix increases the pressure acting on the fluid. This type of Donnan theory has been used to interpret data obtained from experiments studying the swelling of brain tissue slices in culture medium when the cells have been damaged by inhibition of cell metabolism [295–297]. Furthermore, it has been asserted that this theory for a gel can be used to interpret the formation of oedema in the brain [295–298].

The Donnan theory with the entire tissue intracellular and extracellular treated like a single gel does provide a plausible description of the swelling of the damaged tissue slices. However, there are several reasons why somewhat more care is required in the application of Donnan theory to interpret the development of ischaemic brain oedema. Firstly, the theory as applied to the slices takes no account of the effects of the cell membranes or the blood–brain barrier, which are important in situ, and in particular the Donnan theory for slices does not allow for differences in the handling of Na^+^ and of K^+^ in the initial stages of the oedema development. Secondly, as a result of damaging the cell membranes in the slices, many of the impermeant solutes, some with negative charge, will escape from the inside of the neurons and astrocytes and be washed away. Much of the oedema in ischaemic brain develops before the cell membranes are completely disrupted and thus the "fixed" charge densities in the swelling brain and in swelling slices will be different. Thirdly, analysis of the slice preparation does not allow for the differences in the behaviour of astrocytes and neurons. Finally, even if the cell membranes are disrupted completely in later stages of oedema, thus exposing the intracellular gel directly to that outside the cells, most of the oedema will be accumulating outside of the relics of the cells in grossly swollen extracellular spaces (often in white matter), i.e. not within what was the cellular gel.

## Abbreviations

ADC: Apparent diffusion constant (in MRI measurements)
ADP: Adenosine diphosphate
AQP4: Aquaporin 4
ATP: Adenosine triphosphate
CCL2: Chemokine ligand 2, monocyte chemoattractant protein-1
*c*_*i*_: Concentration of ith solute
CNS: Central nervous system
CPP: ( ±)-3-(2-carboxypiperazin-4- yl)-propyl-1-phosphonic acid
CSF: Cerebrospinal fluid
4-CIN: 4-hydroxycinnamate
DHO: Dihydro-ouabain
DIDS: 4,4 -diisothiocyanatostilbene-2,2 -disulphonic acid
DNQX: 6,7-dinitroquinoxaline-2,3-dione
DOTA-Gd: Gadoteric acid (Dotarem®)
ECF: Extracellular fluid
*F*: The Faraday constant
ICF: Intracellular fluid
*ICP*: Intracranial pressure
IMP^−^: Impermeant solutes with net negative charge
ISF: Interstitial fluid
K_ATP_: ATP sensitive K^+^ channel
KCa3.1: Intermediate-conductance calcium-activated K + channel 3.1, IKCa1
KCC2: K^+^, 2Cl^−^ cotransporter 2
Kir4.1: Potassium channel with subunit type 4.1 of the inwardly rectifying potassium channel family
MCAO: Middle cerebral artery occlusion
MCT2: Moncarboxylate transporter 2
MRI: Magnetic resonance imaging f
NAD^+^: Nicotine adenine dinucleotide, deprotonated
NADH: Nicotine adenine dinucleotide, protonated
NBCe1: Na^+^, 2 HCO3^−^ cotransporter 1
NHE: Na^+^/H^+^ exchanger
NKCC1: Na^+^, K^+^, 2Cl^−^ cotransporter 1
ODN: oliodeoxynucleotides
Pi: Phosphate
*R*: Universal gas constant
siRNA: Small interfering RNA
SUR1-TPRM4: Sulfonylurea receptor 1-Transient receptor potential melastatin 4
*T*: Absolute temperature
TBOA: DL-threo-b-Benzyloxyaspartic acid
TEER: Transendothelial electrical resistance
tPA: Tissue plasminogen activator
TRAM-34: 1-[(2-chlorophenyl)diphenylmethyl]-1H- pyrazole [299]
TREK-1: A two-pore domain K^+^ channel, K2p 2.1
TTX: Tetrodotoxin
TWIK-1: A two-pore domain K^+^ channel,, K2p 1.1
∆V_m_: Cell membrane potential
ϕ: Fraction of a porous solid occupied by the pores (or interstitial spaces)

## Footnotes

1 "Oedema" has implied *tissue* swelling for more than a century. With this definition, a simple redistribution of water between cells and ISF does not qualify as a type of oedema [7]. Simard and coworkers [18–21]) have proposed redefinition of the word oedema so that it no longer implies *tissue* swelling. In their notation, cell swelling, even when it occurs entirely at the expense of ISF, is cytotoxic oedema while what has traditionally been called cytotoxic oedema becomes a superposition of cytotoxic and ionic oedema. One possible motivation for this change is to allow different names for two different sets of molecular processes, one occurring in parenchymal cells, the other in the endothelial cells of the blood–brain barrier. It has been suggested that such a change in nomenclature allows increased precision and specificity in descriptions of the processes. However, anything that can be said using one notation could also be said using the other. "Cellular oedema" is just "cell swelling" from the traditional terminology while "ionic oedema" refers to "the concept of transcapillary water and electrolyte transport into brain parenchyma" [18]. Ionic oedema as a concept is not something that can be observed. It simply restates that cytotoxic cell swelling only becomes cytotoxic oedema (old nomenclature) when there is influx of material from outside. This review follows Klatzo and Betz in referring to “cell swelling” with “oedema” reserved for swelling of the tissue. Their nomenclature has a number of advantages:

- it maintains the connection between the meaning of "oedema" in discussions of mechanisms and the meaning of it in clinical practice where "oedema" implies macroscopic, i.e. tissue swelling;
- it avoids having different meanings for "oedema" when referring to the brain or to peripheral tissues;
- it avoids the incorrect implication that "ionic oedema" is a separate type of swelling that has been observed in the absence of "cytotoxic oedema" (new notation); and
- it avoids the implication that the osmotically active solutes added to the parenchyma have to be ions, making it easier to discuss the role of osmotically active metabolites.

In the usage adopted here "cellular oedema" means oedema (i.e. swelling of a tissue) in which most of the extra volume is located within the cells and "extracellular oedema" means oedema in which most of the extra volume is in the extracellular fluid (compare usage in [22]). The term "cell swelling" carries no implication for whether or not the tissue is swollen.

2 Blood-flow in the penumbra is greater than that in the core and, at least initially there is not the depolarization seen in the core but nevertheless electrical activity is absent. A number of factors may contribute to this loss of activity with a likely cause being suppression of release of excitatory transmitters, prominently glutamate, which in turn may be related to changes in ATP and Ca^2+^ levels in presynaptic terminals [29, 33, 37, 43].

3 Siesjö et al. [50] draws attention to the importance of the ATP/ADP ratio rather than the ATP concentration itself as the factor driving activity in the cell, which includes that of the Na^+^-pumps. The ratio can fall to low levels even though the ATP concentration remains a substantial fraction of the normal value. Reference to the consequences of low ATP levels in this review should more properly be to the consequences of low ATP/ADP ratios.

4 Spreading depolarization is now the preferred name for Leão’s spreading depression [71] because the wave of depolarization can be seen to propagate through regions of tissue in which nervous activity is already depressed. The depolarization does suppress activity but it is not necessary for there to be activity that can be suppressed for the depolarization to propagate. Some authors, see e.g. [56], refer to both the initial depolarization in the core and the disturbance that propagates as spreading depolarization.

5 The depolarization at any location is ended if and when the transporters and channels mediating the inward Na^+^ currents inactivate or desensitize sufficiently to allow the Na^+^-pump to extrude Na^+^ restoring the concentration gradients and potential (see Fig. 3). In the core this is not possible because ATP levels are too low, but in the surrounding well perfused tissue and initially in the penumbra, the depolarization can be reversed. In healthy tissue, blood flow is increased, partially via vasodilatation linked to production of NO, and sufficient [glucose] and pO_2_ are achieved in the cells to regenerate ATP to original levels. However, in the penumbra where blood flow is already compromised, there is instead a vasoconstriction linked to the increase in [K^+^]_isf_ which further reduces supply of O_2_ and glucose and slows the recovery [38, 48, 72–79]. (For extensive discussion of the vascular responses see [60]). It is thought that each depolarization leads to an expansion of the size of the core at the expense of the penumbra [80, 81] possibly via decreases in [glucose] and increases in [lactate^−^] [82].

6 If instead ATP levels remain adequate, Na^+^ transported inwards by NBCe1 (see Fig. 7) is transported back out in partial exchange for K^+^ by the Na^+^-pump, which is stimulated by the increase in [K^+^]_isf_ (see discussion in [96]). Because the pump carries net positive charge outwards it will also tend to repolarize the astrocyte allowing more K^+^ entry via the conductance pathway.

7 When K^+^ enters astrocytes in the context of localized neuronal activity it can be removed by current flow through the astrocyte and K^+^ efflux at distal sites, a process called spatial buffering. This process is an alternative to entry of anions. However, spatial buffering cannot account for the extensive uptake and retention of K^+^ when entry occurs over an extended region as in ischaemia [96, 99, 100].

8 Decreased ISF volume can been seen in experiments recording: a marked increase in the impedence of the tissue [43, 101, 102]; an increase in concentration of an extracellular marker [103]; reduced space available for the diffusion of an extracellular marker [104–107]; and, using diffusion weighted MRI imaging, a decrease in the apparent diffusion constant (ADC) of water. The ADC decreases as water moves from ISF where the diffusion constant is relatively large to the cells where the diffusion constant is relatively small [73, 106, 108–110]. ADC measurements have the advantages that they are non-invasive and can be repeated at intervals. The ADC can remain reduced for days as the oedema develops [111]. The fluid removed from the extracellular space is taken up by neurons and astrocytes (see Sects. 3.1 and 3.3 respectively).

9 It should be noted that even though the volume increase requires that fluid enters either from CSF or from the blood this is not vasogenic oedema. Vasogenic oedema results from a malfunction or breakdown of the blood–brain barrier whereas here the ion entry occurs via the normal physiological mechanisms responding to the altered ISF concentrations of Na^+^, K^+^ and Cl^−^ and the production of osmotically active metabolites within the parenchymal cells.

10 Mestre et al. [26] have investigated the very early responses after MCAO produced in mice by embolism with macrospheres. This technique has the advantage of allowing obstruction of the circulation in the region supplied by the middle cerebral artery at an accurately known time after injection of CSF markers and radiotracers into the cisterna magna. It should also be noted that it may produce less disturbance of perivascular transport than other techniques (see [2]).

11 Using measurements of the movement of the brain surface in cats, Hossmann [114] had previously observed swelling within a few minutes of MCAO. However, presumably because it was so rapid, he proposed that the swelling was a consequence of "autoregulatory dilation of the vascular bed (hypoperfusion hyperemia)", i.e. that it was not oedema. However, he did not measure the blood volume and indeed none of the evidence he presented contradicts the alternative idea that the swelling was due to an influx of CSF.

12 For a normal blood flow per gram wet weight of tissue, 540 µL g^−1^ min^−1^ [126], a ratio of wet to dry weight of 5, and a restriction of blood flow to 1% of its normal value, the flow per gram dry weight is still 1.6 mL g^−1^ h^−1^ which can be compared with rates of fluid gain in ischaemic tissues of ~ 0.2 mL g^−1^ h^−1^ (see Table 1). Thus over most of the volume of parenchyma affected by ischaemia, including much of the core, transfer of fluid from blood to brain will be far less than the rate at which fluid is delivered by the residual blood flow.

13 The size of the effect of decreasing [Na^+^]_isf_ on passive Na^+^ net flux can be estimated from the measured permeability surface area product, *PS*, for Na^+^, approximately 0.074 µL g^−1^ h^−1^ [144, 145] where the surface area has been calculated per gram wet weight of tissue. This becomes 0.37 µL g^−1^ h^−1^ when referred to dry weight. For an 80 mM decrease in [Na^+^]_isf_ [43] and thus for a difference in concentration of this size across the blood–brain barrier, the prediction is a reduction in efflux from ISF and increase in net flux into ISF of 30 µmol g^−1^ h.^−1.^

14 The interpretation of the inhibitor results is not entirely straight-forward because these inhibitors can also act on cells within the parenchyma ([156–158] and for discussion [142, 147, 159]). However, ouabain and, as pointed out by O'Donnell [146] bumetanide do not cross the blood–brain barrier into the brain (see [160, 161] and Footnote 5 in [142]) and therefore when applied to the blood-side of the barrier their effects on production of oedema must be via actions on transporters on brain endothelial cells.

15 Leakage of serum albumin has been seen to correlate directly with the formation and resolution of "pure" vasogenic oedema. In this there is no damage to cells within the parenchyma but blood vessels are temporarily made permeant to large and small solutes [177] (see also [178]). However, those are not the conditions that exist with ischaemic oedema.

16 Glibenclamide also inhibits K_ATP_ channels by binding to their SUR2 subunits. However, it is believed that it is the inhibition of SUR1-TRPM4 rather than K_ATP_ that is more important in explaining the effect of glibenclamide on oedema formation. K_ATP_ channels are K^+^ selective. During the development of oedema, block of K^+^ selective channels would prevent K^+^ loss and increase Cl^−^ gain accompanying Na^+^ entry (see Sect. 3), i.e. it would increase the rate of NaCl accumulation and cell swelling rather than reducing the rate as noted above. The relative importance of block of SUR1-TRPM4 channels is also supported by results showing that antisense knockdown of SUR1-TRPM4 channels (antisense oligodeoxynucleotides (ODNs) against either Abcc8/SUR1 or Trpm4/TRPM4) significantly reduce swelling while antisense knockdown of K_ATP_ (antisense-ODNs, either Kcnj8/Kir6.1 or Kcnj11/Kir6.2) have no effect [186]. Assuming that the Kir ODN's have similar access to the cells in which the anti-SUR1 and anti-TRPM4 ODNS are effective these results suggest that glibenclamide's effects on oedema can be ascribed to its blockade of SUR1-TRPM4 channels, plausibly those located on the endothelial cells of the blood–brain barrier.
